# Notch1 O-GlcNAcylation drives tumor stemness and mechanoadaptation to a stiff microenvironment and promotes chordoma recurrence

**DOI:** 10.1172/JCI194378

**Published:** 2026-02-03

**Authors:** Chengjie Lian, Weiyan Peng, Peiqiang Su, Yan Ye, Jialing Liu, Dongsheng Huang, Xuejuan Sun, Yi Pu, Zhiheng Liao, Xudong Wang, Zhu Qiu, Shanshan Wu, Lei Liu

**Affiliations:** 1 Department of Breast and Thyroid Surgery, Chongqing Key Laboratory of Molecular Oncology and Epigenetics, The First Affiliated Hospital of Chongqing Medical University, Chongqing, China.; 2 Department of Orthopaedics, Sports Injury Division, Fujian Medical University Union Hospital, Fuzhou, Fujian, China.; 3 Department of Orthopedics and; 4 Guangdong Provincial Key Laboratory of Orthopedics and Traumatology, The First Affiliated Hospital of Sun Yat-sen University, Guangzhou, Guangdong, China.; 5 Department of Spine Surgery, Sun Yat-sen Memorial Hospital, Sun Yat-sen University, Guangzhou, Guangdong, China.; 6 Department of Biology, School of Basic Medical Science, Guangdong Medical University, Zhanjiang, Guangdong, China.

**Keywords:** Cell biology, Oncology, Cancer, Cytoskeleton, Extracellular matrix

## Abstract

Chordomas are rare malignant osseous neoplasms with a striking rate of recurrence. Primary chordomas typically originate from embryonic notochord remnants, whereas recurrent chordomas usually stem from tumor cells infiltrating bone or cartilage after surgery. Clinically, the recurrent chordomas exhibit a stiffer extracellular microenvironment (ECM) than primary tumors. Intriguingly, this study identified cytoskeleton rearrangement, stress fiber reorganization, enhanced stemness, and Notch signaling activation in recurrent chordoma tissues or cell lines surviving stiff substrates, indicating the critical roles of mechanical remodeling and tumor stemness in stiffness resistance. We propose a potentially novel recurrence model where tumor cells experience mechanoadaptive organization, which enables them to resist stiff microenvironment-induced cell death. O-GlcNAcylation of Notch1 intracellular domain (NICD1) is central to this process. Mechanistically, the stiff ECM-driven ligand-independent phosphorylation of EPHA2 sequentially activated LYN kinase and subsequently triggered O-linked N-acetylglucosamine (O-GlcNAc) transferase (OGT) activity by phosphorylating Y989 and Y418, critical residues for OGT glycosyltransferase activity; this induced NICD1 O-GlcNAcylation at T2063, T2090, and S2162, specifically promoting transcription of mechanical and stemness-related genes. *MIR31* deletion upregulated LYN, enhancing stiffness perception and promoting O-GlcNAc addition to NICD1, finally resulting in mechanoadaptation- and tumor stemness–driven recurrence. Consequently, *MIR31* deletion is a potential biomarker for recurrence and patient stratification in Notch- or OGT-targeted therapies.

## Abstract 2

Chordomas acquire stiffness-tolerance through Notch1 O-GlcNAcylation-mediated mechanoadaptation and stemness properties. *MIR31* loss serves as a recurrence biomarker, guiding OGT/Notch-targeted therapeutic strategies.

## Graphical abstract

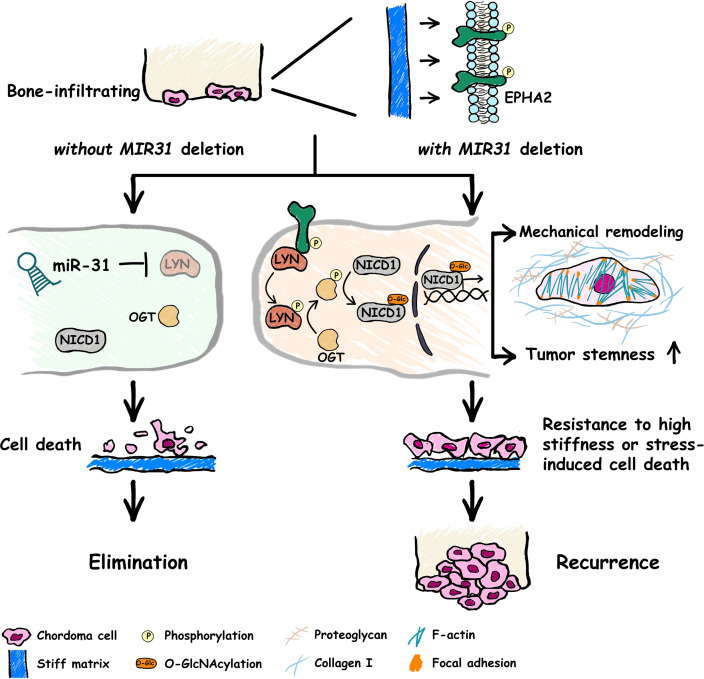

## Introduction

Chordomas are extremely rare malignant bone tumors with an incidence of approximately 1:1,000,000 cases per year, which accounts for 1%–4% of all bone malignancies (1, 2). Owing to their insidious onset, delayed growth, and deep anatomical location, the tumors are often not diagnosed until enlarged and are accompanied by severe bone pain, spinal instability, and nerve compression (2, 3). The challenges in complete resection, and resistance to conventional therapies, deem chordoma eradication difficult (4, 5). Despite efforts to employ tyrosine kinase inhibitors and target small molecules against the brachyury transcription factors, the efficacy of these treatments remains to be empirically validated in clinical settings (2, 6–8). Over 60% of patients with chordoma experience relapse after initial treatment (2, 9). The high recurrence rate and lack of effective therapeutic strategies necessitate the exploration of its pathogenesis and the development of innovative therapeutic approaches.

As observed clinically, primary chordomas originate from remnants of primitive embryonic notochord tissue and are characterized by soft, jelly-like properties, whereas recurrent chordomas arise from tumor cells infiltrating the bone, cartilage, and surrounding tissues with higher stiffness, posing challenges for surgical clearance (10–13). Consequently, the mechanical properties of the microenvironment differ substantially between primary and recurrent tumors. Comprehending the mechanism underlying the adaptation of chordoma cells to the stiff microenvironment of recurrence is crucial. Tumor mechanical properties are “dynamic and adaptive” and usually display extracellular microenvironment (ECM) and cellular remodeling, such as regulation of collagen and proteoglycan expression, cytoskeleton rearrangement, stress fiber reorganization, and so on (14–18). Therefore, whether and how ECM and cellular mechanical remodeling mediate tumor cell adaptation to the recurrent microenvironment, thereby promoting chordoma recurrence, warrants further investigation.

Cancer stem cells (CSCs) represent a subpopulation of tumor cells with the ability to self-renew and differentiate into heterogeneous lineages, contributing to metastasis, recurrence, and therapy resistance (19, 20). The mechanism underlying their dysregulation varies across cancer types, and targeting CSCs is a potential therapeutic strategy (19, 21–23). Our previous studies highlighted the critical role of chordoma stem cell in relapse and proposed potential biomarkers for identifying drug-sensitive populations and novel treatment strategies (24). In the current study, we revealed Notch activation in recurrent tumors, which correlates with the signatures of mechanical remodeling and CSCs. While negative regulation normally restrains the overactivation of Notch signaling under physiological conditions, the mechanism underlying Notch signaling activation in recurrent tumors remains unclear (25).

Glycosylated proteins not only serve as important biomarkers for tumor diagnosis, but also participate in tumorigenesis and cancer progression, offering novel therapeutic targets (26). O-linked N-acetylglucosamine (O-GlcNAc), an atypical glycosylation modification, has been implicated in the regulation of various cytosolic and nuclear oncogenic and tumor-suppressive proteins. Aberrant O-GlcNAcylation in oncogenic regulators like c-MYC and p53 has been shown to modulate protein stability, localization, and transcriptional activity in response to upstream cues such as nutrient availability and inflammatory signals (26–29). We observed upregulation of Notch1 intracellular domain (NICD1) O-GlcNAcylation in response to increased ECM stiffness, with a positive correlation between NICD1 O-GlcNAcylation and Notch activation, mechanical properties, and tumor stemness. Notch activation depends on the cleavage between the Notch1 extracellular domain (NECD1) and the intracellular domain, which releases NICD1 for translocation to the nucleus. Previous studies have demonstrated that O-GlcNAcylation of EGF-like repeats of NECD1 enhances cell proliferation and EMT (27, 30). The dysregulation of O-GlcNAc transferase (OGT) and O-GlcNAcase–mediated (OGA-mediated) aberrant O-GlcNAcylation has been implicated in cancers (31). Therefore, elucidating whether and how NICD1 O-GlcNAcylation transduces ECM mechanical signals into intracellular biological signals, thereby initiating mechanical remodeling to mediate tumor adaptation to a stiff microenvironment, warrants further investigation.

## Results

### Remodeling of mechanical properties and increased tumor stemness were identified during chordoma recurrence.

**Figure 1:**
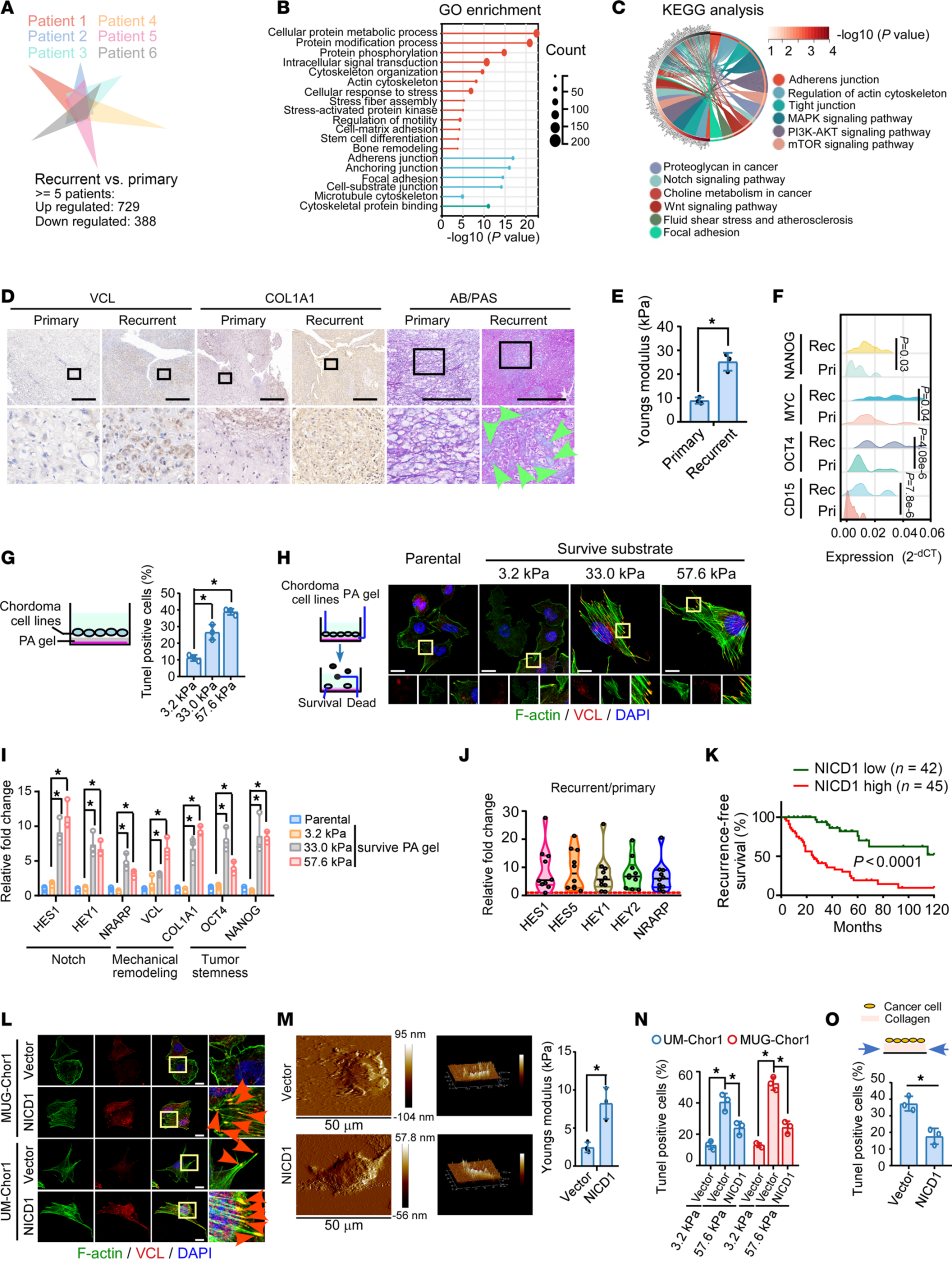
Mechanical properties of remodeling and increased tumor stemness were identified during chordoma recurrence. (**A**–**C**) We performed mRNA microarray analysis on 6 matched primary-recurrent tumor pairs. Genes that were differentially expressed in at least 5 of these pairs were subsequently analyzed by GO enrichment and KEGG analysis. (**D**) IHC was performed to determine VCL and COL1A1 expression levels in chordoma tissues. Alcian blue/periodic acid–Schiff’s (AB/PAS) staining was utilized to detect proteoglycan levels. Green arrows show proteoglycan. Scale bar: 500 μm. (**E**) Young’s modulus of primary and recurrence chordoma tissues, as detected by using atomic force microscopy (AFM). (**F**) qRT-PCR was used to assess the expression of CSC-related genes in 9 paired primary and recurrent chordoma tissues. (**G**) Chordoma cells were cultured on PA gel substrates with different stiffnesses for 48 hours. The TUNEL assay was used to detect the effect of increased stiffness on cell death. (**H** and **I**) In tumor cells surviving high stiffness, phalloidin staining and IF were used to determine F-actin and VCL expression levels, respectively. (**H**) The colocalization areas indicate focal adhesions. Scale bar: 10 μm. (**I**) Expression of Notch signaling–, mechanical properties–, and tumor stemness–related genes were analyzed using qRT-PCR. (**J**) qRT-PCR was used to assess the expression of Notch signaling downstream genes in 10 paired primary and recurrent chordoma tissues. (**K**) Kaplan-Meier analysis of the recurrence-free survival of a cohort of 87 patients with chordoma using the log-rank test. NICD1 expression was determined using IHC, and the median value was used as the cut-off value. (**L**) Phalloidin staining and IF were used to assess the effects of the NICD1 ectopic expression on cytoskeleton. Red arrows show focal adhesions. Scale bar: 10 μm. (**M**) AFM determines Young’s modulus of cells ectopically expressing NICD1. (**N**) The effects of NICD1 overexpression on stiff substrate-mediated apoptosis were analyzed. (**O**) TUNEL assay results of cells cultured on type I collagen with periodically stress stimulation. The pressure parameter is set to 10% compression rate (achieve compression rate in 1 second, hold for 1 second, and then return to the initial state in 1 second) for a cumulative duration of 3 hours. TUNEL staining was performed at 48 hours. Data in **J** are presented as mean (minimum to maximum). Data in **E**, **G**, **I**, and **M**–**O** are presented as mean ± SD. Statistical analysis was performed using unpaired Student’s *t* test (**E**, **M**, and **O**) and 2-way ANOVA (**G**, **I**, and **N**). *P < 0.05.

As observed clinically, the mechanical properties of the microenvironment differ significantly between primary and recurrent tumors. The ECM of recurrent tumors usually exhibits significantly increased stiffness. Here, we investigated whether and how chordoma cells adapt to stiff microenvironments. The mRNA microarray assay, coupled with Gene Ontology (GO; https://geneontology.org/) and Kyoto Encyclopedia of Genes and Genomes (KEGG; https://www.genome.jp/kegg/) enrichment analyses in 6 pairs of primary and recurrent chordoma tissues, revealed that differentially expressed genes in recurrent tumor cells were associated with ECM remodeling, cytoskeleton rearrangement, and stress fiber reorganization (Figure 1, A–C). Furthermore, downregulated mucus secretion and upregulated COL1A1, vinculin (VCL), focal adhesion, proteoglycan, and cell stiffness were observed during recurrence (Figure 1, D and E). These findings indicated the mechanoremodeling of tumor cells. Interestingly, in some patients with chordoma, the primary tumor was mucus enriched and the cells were arranged in a cord-like pattern, whereas the recurrent tissues displayed cell-enriched, poorly differentiated, undifferentiated, or even mixed types (Supplemental Figure 1A; supplemental material available online with this article; https://doi.org/10.1172/JCI194378DS1). The upregulation of tumor stemness–related genes was observed in recurrent chordomas (Figure 1F). Therefore, we speculated that mechanoremodeling and the accumulation of tumor stemness might participate in chordoma recurrence.

To explore the effects of a high-stiffness microenvironment on chordoma cell growth, polyacrylamide (PA) hydrogel substrates of varying stiffness were employed. As reported, embryo notochord tissue displays soft jelly-like properties (<2–4 kPa), whereas recurrent chordomas originate from infiltrating tumor cells facing a stiffer microenvironment, including bones (usually >50 kPa) (10–13). Thus, a gradient of stiffness ranging from 3.2 kPa to 57.6 kPa was adopted in this study. We found that chordoma cells cultured on substrates mimicking high stiffness (~57.6 kPa) showed growth inhibition and increased apoptosis compared with those cultured on softer substrates (~3.2 kPa) (Figure 1G and Supplemental Figure 1, B and C). Impressively, chordoma cells surviving high stiffness exhibited mechanical remodeling, such as cytoskeleton rearrangement, stress fiber (F-actin bundle) organization, increased focal adhesion, and VCL and COL1A1 expression (Figure 1, H and I). These findings suggest a resistance mechanism against high stiffness–mediated growth inhibition in recurrent tumors, possibly contributing to tumor cell survival.

Among the stemness-related pathways, Notch signaling was correlated with chordoma relapse (Figure 1C). Interestingly, markedly increased Notch signaling activity, along with tumor stemness, was observed in chordoma cells that survived high stiffness (Figure 1I). Upregulated expression of Notch signaling downstream genes and increased accumulation of nuclear Notch1 were observed in recurrent tumors compared with the corresponding primary tumors (Figure 1J and Supplemental Figure 1D). Remarkably upregulated Notch signaling activity was observed in CD15^+^ tumor cells from patients with chordoma (Supplemental Figure 1E). As observed in the current study, patients with chordoma with relatively high Notch signaling activity had a shorter recurrence-free survival time than those with lower Notch signaling activity (Figure 1K and Supplemental Tables 1–3). To further probe the findings, we constructed a recurrent chordoma mouse model, and MUG-Chor1-pri and MUG-Chor1-rec cell lines were generated (Supplemental Figure 1, F and G). A growth dominance of MUG-Chor1-rec cells relative to MUG-Chor1-pri cells was established in vivo (Supplemental Figure 1, H and I). Remarkably upregulated Notch signaling was observed in tumors derived from MUG-Chor1-rec cells (Supplemental Figure 1J).

We further explored whether Notch signaling participates in chordoma mechanical remodeling and stemness acquisition. Ectopic expression of NICD1 in chordoma cell lines potentiated their ability to grow into larger, more numerous, nonadherent cell spheres; increased the proportion of CD15^+^ cell fractions; and decreased their sensitivity to cisplatin (Supplemental Figure 1, K–M, and Supplemental Table 4). As shown in Supplemental Figure 1, N and O, mice subcutaneously inoculated with MUG-Chor1-NICD1 cells had shorter tumor-free survival times and developed larger tumors. Notch activation promoted stress fiber reorganization and increased VCL, focal adhesion, and cell stiffness, indicating the remodeling of cellular mechanical properties (Figure 1, L and M). Interestingly, the phenomenon of cell death induced by a stiff substrate or high stress could be partly reversed by the overexpression of NICD1, indicating that Notch signaling activation might allow chordoma cells to adapt to a stiff microenvironment (Figure 1, N and O). We analyzed the expression profiles of glioma, another nervous system tumor, and sarcoma, another malignant tumor originating from the mesoderm, and found that the expression of aggressiveness or mechanical property-related genes was much higher in patients bearing tumors with Notch signaling activation (Supplemental Figure 1, P and Q). Taken together, these findings highlight the critical role of aberrant Notch signaling in mediating tumor mechanoadaptive rearrangement and CSC-like properties during chordoma recurrence.

### Increased NICD1 O-GlcNAcylation contributes to the aberrant activation of Notch signaling in recurrent chordoma.

**Figure 2:**
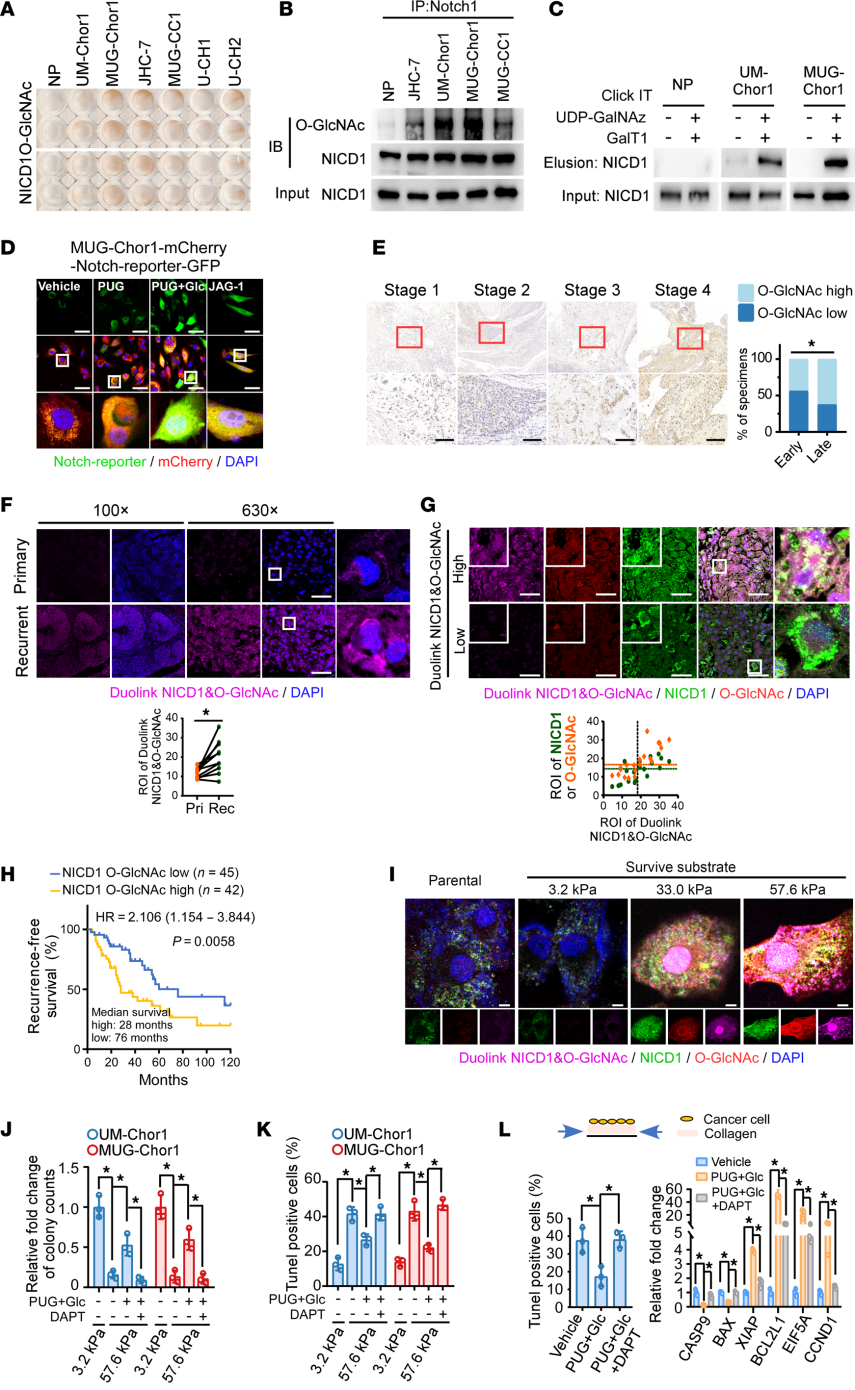
Increased NICD1 O-GlcNAcylation contributes to aberrant Notch signaling activation in recurrent chordoma. (**A**) IHC was used to assess levels of global O-GlcNAc and NICD1 in established NP and chordoma cell lines. (**B**) IP was used to analyze NICD1-specific O-GlcNAcylation levels. (**C**) Detection of NICD1 O-GlcNAcylation by the Click-iT O-GlcNAc Enzymatic Labeling System. (**D**) GFP to assess the effect of PUGNAC (50 μM, 24 hours) and GlcNAc (10 μM, 24 hours) treatment on Notch signaling activity. JAG-1 (40 μM, 24 hours) was used as the positive control in activating Notch signaling. Scale bar: 50 μm. (**E**) IHC was used to assess expression of O-GlcNAc expression in early- (*n* = 55) and late-stage (*n* = 32) chordoma. Scale bar: 100 μm. (**F** and **G**) Duolink PLA was used to assess NICD1-specific O-GlcNAc in clinical specimens (**F**, *n* = 10 pairs). NICD1 and global O-GlcNAc expression was detected using IF assay, and the correlation between global O-GlcNAc expression and NICD1-specific O-GlcNAc expression was analyzed (**G**, *n* = 20). Scale bar: 50 μm. The black dotted line shows the median value of Duolink, while the green and orange lines indicate the median values of NICD1 and global O-GlcNAc, respectively. (**H**) Kaplan-Meier analysis of recurrence-free survival. The NICD1-specific O-GlcNAc level was assessed using Duolink PLA in specimens. Median survival time and horizon risk (HR) of recurrence were listed. (**I**) Duolink PLA detects NICD1-specific O-GlcNAc in chordoma cells surviving substrates with different stiffnesses at 48 hours. Scale bar: 1 μm. (**J** and **K**) Colony-formation and TUNEL assay results from cells cultured on substrates with different stiffnesses and indicated treatment. Cells were cultured and treated with PUGNAC (50 μM), GlcNAc (10 μM), and DAPT (20 μM) for 7 days (**J**) or 48 hours (**K**). (**L**) TUNEL assay and qPCR results from cells cultured on collagen, with periodic stress stimulation to determine the effects on cell death (as described in the legend for Figure 1O). Data in **J**–**L** are presented as mean ± SD. Statistical analysis was performed using χ^2^ test (**E**), paired Student’s *t* test (**F**), and 1-way ANOVA (**J**–**L**). *P < 0.05.

Next, we explored the mechanism underlying the activation of Notch signaling in chordomas. The established chordoma cell lines exhibited substantially higher levels of global O-GlcNAcylation and activated NICD1 than human primary nucleus pulposus (NP) cells, with a positive correlation between NICD1 and global O-GlcNAcylation levels (Figure 2A and Supplemental Figure 2A). Previous studies have demonstrated O-GlcNAcylation of EGF-like repeats in NECD1 (27). We investigated whether the released NICD1 could also be O-GlcNAcylated. In support of our hypothesis, the YinOYang 1.2 Server identified potential O-GlcNAcylation residues in NICD1, and NICD1 O-GlcNAcylation was confirmed by IP and the Click-iT O-GlcNAc Enzymatic Labeling System (Invitrogen) and the Click-iT Protein Analysis Detection Kit (Figure 2, B and C, and Supplemental Figure 2B).

Overexpression of OGT and treatment of UDP-GlcNAc, the donor substrate for GlcNAc, markedly increased NICD1 O-GlcNAcylation (Supplemental Figure 2C). In parallel, remarkable accumulation of global O-GlcNAcylation, NICD1-specific O-GlcNAcylation, and Notch signaling activity was observed when chordoma cells were incubated with GlcNAc and PUGNAC, an inhibitor of O-GlcNAcase (OGA) that removes O-GlcNAc modifications (Supplemental Figure 2, D–F). We also constructed MUG-Chor1 cells that stably expressed mCherry and the Notch signaling reporter GFP, which provided an indicator for assessing the activation level of Notch signaling. Figure 2D shows that the combination of GlcNAc and PUGNAC led to a substantial accumulation of GFP fluorescence, akin to canonical Notch pathway activation by JAG-1. Clinically, the positive correlation between glycosylation and Notch activity was validated by GSEA in gliomas and sarcomas (Supplemental Figure 2G). These results established the robust capacity of O-GlcNAcylation for Notch activation.

Our findings revealed that OGT is a binding partner of NICD1 (Supplemental Figure 2, H and I). Repression of NICD1 O-GlcNAcylation and Notch signaling activity was observed in chordoma cells incubated with OSMI-1 or DON, which are inhibitors of OGT glycosyltransferase activity (Supplemental Figure 2, J–M).

Clinically, we observed higher global O-GlcNAcylation levels in late-stage patients with chordoma than in early-stage patients (Figure 2E), with increased O-GlcNAcylation, NICD1-specific O-GlcNAcylation, NICD1, and aggressiveness-related genes in recurrent tumors compared with primary tumors in clinical and in vivo studies (Figure 2, F and G, and Supplemental Figure 2, N and O). Moreover, a positive correlation between Notch signaling activity and NICD1-specific O-GlcNAcylation levels was noted clinically (Supplemental Figure 2, N and P). Patients with higher NICD1-specific O-GlcNAcylation levels experienced earlier relapse (Figure 2H and Supplemental Table 5). Importantly, increased NICD1-specific O-GlcNAcylation was observed in chordoma cells surviving high stiffness (Figure 2I). Pro–O-GlcNAcylation–mediated resistance to apoptosis induced by high stiffness or stress could be abrogated by DAPT (a Notch signaling inhibitor) (Figure 2, J–L, and Supplemental Figure 2Q). Therefore, we propose that NICD1 O-GlcNAcylation–mediated Notch signaling activation promotes chordoma recurrence by counteracting the suppression of survival induced by a stiff microenvironment.

### NICD1 O-GlcNAcylation promotes tumor mechanical properties remodeling and tumor stemness.

**Figure 3:**
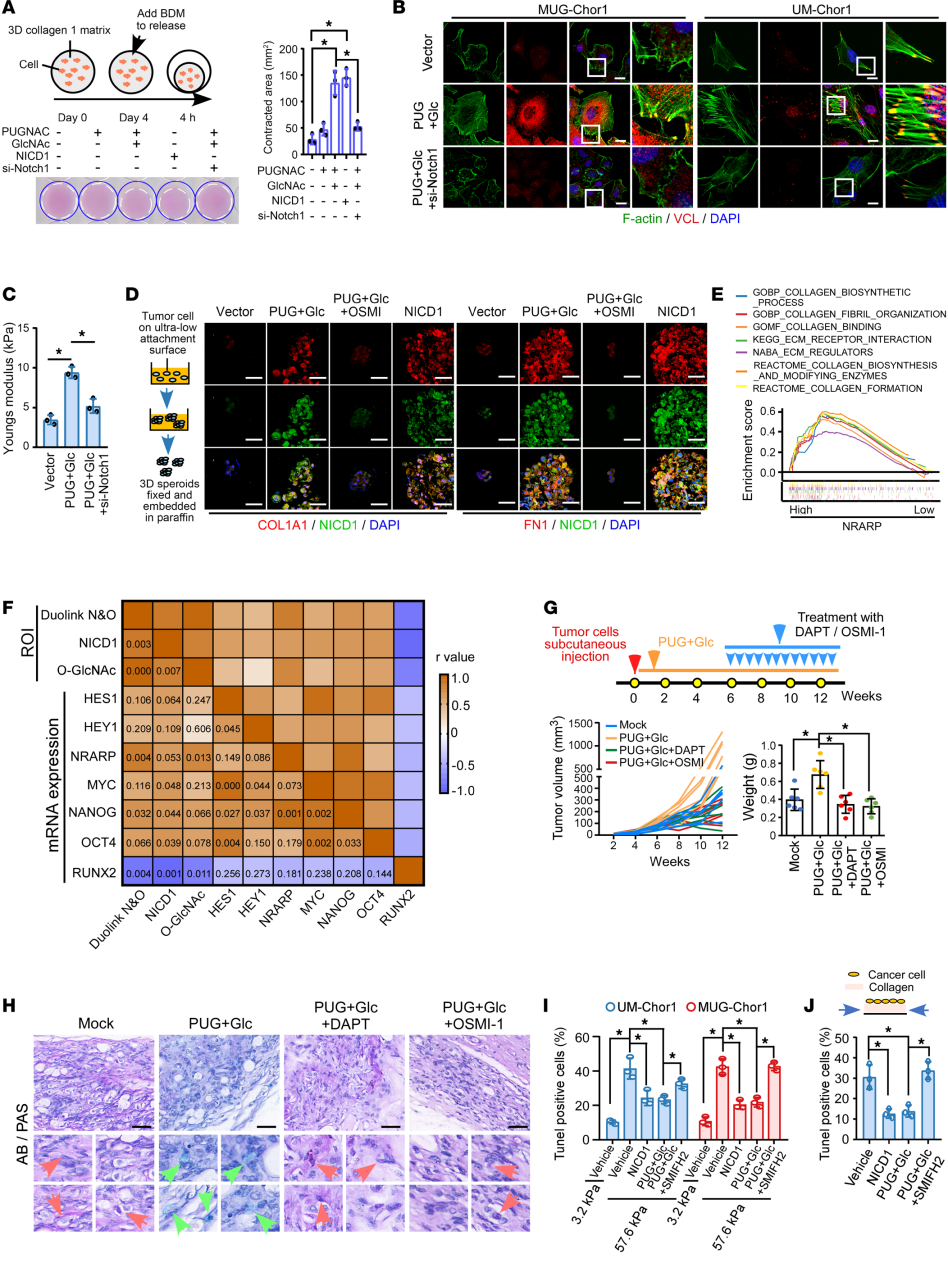
NICD1 O-GlcNAcylation promotes tumor mechanical properties remodeling and tumor stemness. (**A**) Collagen-based cell contraction assay was used to assess cellular contractility and cell-ECM interactions for cells with PUGNAC, GlcNAc, and NICD1 overexpression. Whether the effect of pro–O-GlcNAc treatment could be abrogated by Notch inhibition was also assessed. (**B**) Phalloidin staining and IF were used to analyze F-actin and VCL expression. The colocalization areas indicate focal adhesions. Scale bar: 10 μm. (**C**) AFM was used to detect the stiffness of MUG-Chor1 cells. (**D**) IF was used to detect COL1A1, FN1, and NICD1 expression in spheroids harvested by performing sphere formation assay. Cells were cultured with PUGNAC (50 μM), GlcNAc (10 μM), or OSMI-1 (50 μM) for 7 days. Scale bar: 50 μm. (**E**) GSEA analysis was used to analyze the correlation between Notch activity and mechanical-related gene signature in patients with glioma from the TCGA dataset. (**F**) Protein levels of NICD1 O-GlcNAcylation, NICD1, and global O-GlcNAc were assessed by Duolink PLA and IHC. Expression of Notch signaling–, CSC-, and differentiation-related genes was determined using qRT-PCR (*n* = 10). Correlation was analyzed using the Pearson’s correlation coefficient. *P* values are listed in the boxes. (**G** and **H**) 1 **×** 10^7^ MUG-Chor1 cells were inoculated subcutaneously. Tumors were treated with PUGNAC (10 mg/kg) and GlcNAc (10 mg/kg). After 6 weeks, tumors were treated with DAPT (1 mg/kg) or OSMI-1 (1 mg/kg) every 3 days. (**G**) Volume and weight of the subcutaneous tumors were detected (*n* = 5/group). (**H**) The effects of pro–O-GlcNAc treatment and OSMI-1 on extracellular proteoglycan level were determined by using AB/PAS staining. Red arrows show acid mucus, and green arrows show proteoglycan. Scale bar: 50 μm. (**I** and **J**) TUNEL assay in cells cultured with indicated treatment. Whether inhibiting cytoskeleton rearrangement could abrogate effects of NICD1 O-GlcNAcylation was analyzed by utilizing SMIFH2 (5 μM, 48 hours), which could inhibit actin polymerization. Data in **A**, **C**, **G**, **I**, and **J** are presented as mean ± SD. Statistical analysis was performed using 1-way ANOVA. *P < 0.05.

Chordoma cells incubated with PUGNAC and GlcNAc or NICD1 overexpression exhibited increased cellular contractility and collagen plug contraction, which could be remarkably abrogated by silencing Notch1, supporting the crucial role of O-GlcNAcylation–induced Notch activation in cytoskeletal contraction capacity and ECM remodeling (Figure 3A). Increased F-actin bundles, VCL, and focal adhesion formation were observed in chordoma cells treated with PUGNAC and GlcNAc, which could be abrogated by Notch1 depletion (Figure 3, B and C). Treatment with PUGNAC and GlcNAc or NICD1 ectopic expression strongly induced the expression of COL1A1 and FN1, the key ECM proteins, which could be suppressed by silencing Notch1 (Supplemental Figure 3A). Similarly, in spheroids developed from the sphere formation assay, we also noticed that pro–O-GlcNAcylation treatment induced the upregulation of COL1A1, FN1, and activated Notch1, which was attenuated by OSMI-1 (Figure 3D). Besides, ChIP assay revealed increased affinity of RBP-Jκ, a crucial cotranscription factor of NICD1, with the *COL1A1* promoter upon pro–O-GlcNAcylation treatment, which was diminished by Notch1 depletion (Supplemental Figure 3B). The GSEA analysis of gliomas also indicated a correlation between Notch activity and ECM regulation (Figure 3E). Furthermore, higher expression of COL1A1 and FN1 was observed in tumors with higher Notch signaling activity in patients with sarcoma and glioma (Supplemental Figure 3C). As shown in Supplemental Figure 3, D and E, chordoma cells with OGT overexpression or pro–O-GlcNAcylation treatment displayed enhanced CSC-like properties, which could be abrogated by either Notch1 silencing or OSMI-1. Moreover, a positive correlation was observed between NICD1 O-GlcNAcylation, NICD1, O-GlcNAc, Notch signaling downstream genes, and tumor aggressiveness-related genes, whereas a negative correlation was noted with RUNX2, a gene with prodifferentiation characteristics (Figure 3F).

In vivo, as shown in Figure 3G, treatment with PUGNAC and GlcNAc resulted in larger tumors, which could be reversed by DAPT or OSMI-1, suggesting that O-GlcNAcylation participates in Notch activation–mediated malignancy. Decreased acidic mucus and increased proteoglycan levels were observed in tumors that developed from chordoma cells treated with PUGNAC and GlcNAc, which were blocked by either DAPT or OSMI-1 (Figure 3H and Supplemental Figure 3F).

We further verified the significance of cytoskeletal remodeling in Notch-mediated apoptosis resistance. Impressively, SMIFH2, an inhibitor of actin polymerization, remarkably attenuated the effects of pro–O-GlcNAcylation on the resistance to high stiffness– or stress-induced apoptosis (Figure 3, I and J, and Supplemental Figure 3, G and H).

In conclusion, these findings indicated the essential role of NICD1 O-GlcNAcylation in promoting adaptation to microenvironment bearing high stiffness or stress by regulating mechanical properties and tumor stemness.

### O-GlcNAcylation of T2063, T2090, and S2162 enhances NICD1 stability and transactivation activity.

We investigated the modulation of NICD1 O-GlcNAcylation by PUGNAC and GlcNAc and found increased O-GlcNAcylation levels in both cytoplasmic and nuclear NICD1, which were reversed by OSMI-1 and DON (Supplemental Figure 4A). The interaction between OGT and NICD1 was evident in both the cytoplasm and nucleus (Supplemental Figure 4B). Previous studies have reported multiple OGT splice variants with unique N-terminal locations and biological functions, including mitochondrial OGT (103 kDa), nucleocytoplasmic OGT (116 kDa), and the short isoform of OGT (78 kDa) (32, 33). As shown in Supplemental Figure 4C, NICD1 predominantly bound to nucleocytoplasmic OGT, which was localized to both the nucleus and cytoplasm, further supporting our findings in Supplemental Figure 4A.

**Figure 4:**
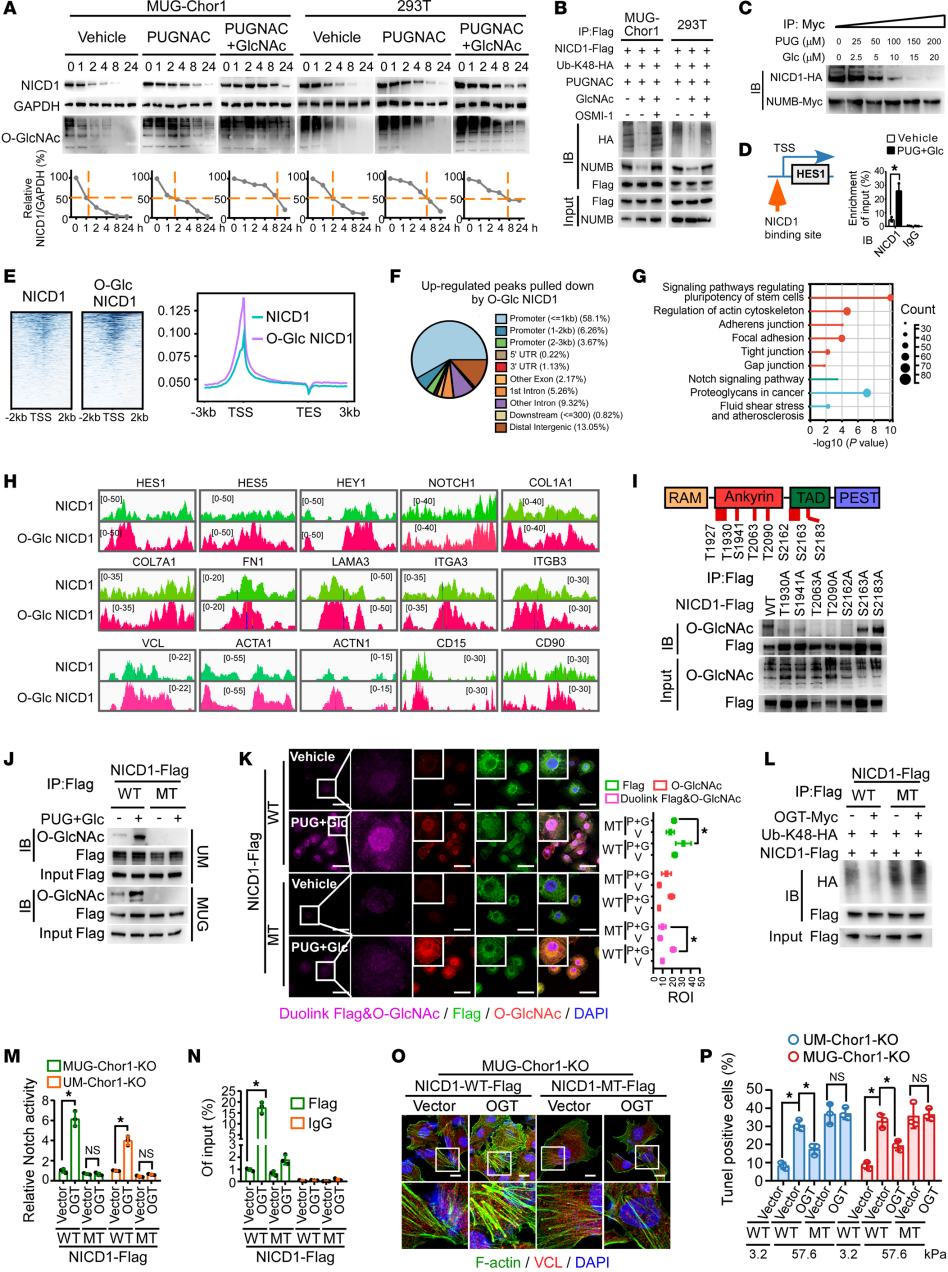
O-GlcNAcylation of T2063, T2090, and S2162 enhances NICD1 stability and transactivation activity. (**A**) WB was used to detect the effect of pro–O-GlcNAc treatment on the half-lives of NICD1 in MUG-Chor1 cells and 293T cells treated with cycloheximide (CHX, 40 μM). (**B**) IP was used to assess the effects of pro–O-GlcNAc and OSMI-1 on the interaction between NUMB and NICD1 and K48-linked polyubiquitination of NICD1. (**C**) Chordoma cells expressing NUMB-Myc and NICD1-HA were treated with incrementally increased PUGNAC and GlcNAc, followed by IP assay to assess the interaction between NUMB and NICD1. (**D**) ChIP analysis was used to identify the enrichment of NICD1 on *HES1* promoter in response to PUGNAC and GlcNAc. (**E**–**H**) ChIP-seq in MUG-Chor1 cells expressing NICD1-Flag with or without PUGNAC and GlcNAc treatment, cell extracts of which were pulled down by Flag. (**E** and **F**) Location and quantification of increased DNA pulled down by O-GlcNAcylated NICD1 compared with NICD1 was analyzed. Enrichment of upregulated genes is shown in **G**. (**H**) The peak on promoter regions of specific genes was shown using IGV. (**I**) IP and MS assay in MUG-Chor1 cells purified by anti-Flag affinity gel to determine the O-GlcNAcylation residues of NICD1. O-GlcNAc of WT and mutant NICD1 was determined. (**J** and **K**) IP and Duolink PLA to determine O-GlcNAcylation of WT and mutant NICD1 (carrying combined mutation of T2063A/T2090A/S2162A) in response to PUGNAC and GlcNAc in MUG-Chor1 cells. Scale bar: 50 μm. (**L**) IP was used to determine the effect of PUGNAC and GlcNAc on ubiquitination of WT and mutant NICD1 in MUG-Chor1 cells. (**M**) Dual luciferase reporter assay was used to detect Notch signaling activity in MUG-Chor1 cells with endogenous NICD1 knockout. (**N**) ChIP assay shows binding of WT and mutant NICD1 to the promoter region of *HES1* in MUG-Chor1 cells. (**O**) Phalloidin staining and IF were used to analyze F-actin, VCL, and focal adhesions in MUG-Chor1 cells with endogenous NICD1 knockout. Scale bar: 10 μm. (**P**) TUNEL assay was used to assess the effect of OGT ectopic expression on WT and mutant NICD1 in chordoma cells with endogenous NICD1 knockout, which were cultured on substrates with different stiffnesses. WT, WT NICD1; MT, T2063A/T2090A/S2162A NICD1. Data in **D**, **K**, **M**, **N**, and **P** are presented as mean ± SD. Statistical analysis was performed using unpaired Student’s *t* test (**D**) and 1-way ANOVA (**K**, **M**, **N**, and **P**). *P < 0.05.

Remarkably, pro–O-GlcNAcylation treatment increased NICD1 protein levels without affecting Notch1 mRNA levels, whereas the inhibition of OGT activity reduced NICD1 protein levels (Supplemental Figure 4, D and E). NICD1 stability is crucial for its nuclear translocation and transcriptional activity. We found that pro–O-GlcNAcylation remarkably enhanced NICD1 stability, which was reduced upon OGT inhibition (Figure 4A and Supplemental Figure 4, F and G). NICD1 accumulation was observed in both the cytoplasm and the nucleus (Supplemental Figure 4H). Notably, O-GlcNAcylated NICD1 exhibited decreased NUMB-mediated ubiquitination and degradation (Figure 4B). Gradient increases in PUGNAC and GlcNAc levels reduced the interaction between NUMB and NICD1 (Figure 4C). Furthermore, pro–O-GlcNAcylation promoted NICD1 affinity to the *HES1* promoter, indicating increased transcription activity (Figure 4D).

ChIP-seq analysis revealed increased transactivation activity of O-GlcNAcylated NICD1, with binding sites predominantly located upstream of the gene-coding regions near the transcription start site (Figure 4, E and F). Notably, O-GlcNAcylated NICD1 tended to target the promoter regions of downstream Notch signaling genes and genes related to chordoma stemness and mechanical properties (Figure 4, G and H).

OGT interacts with the N-terminal tail containing the ANK and TAD domains of NICD1 (Supplemental Figure 5A). Mass spectrometry (MS) revealed that T1927, T1930, S1941, T2063, T2090, S2162, S2163, and S2183 were potential O-GlcNAcylation residues (Figure 4I and Supplemental Figure 5B). Interestingly, these serine and threonine sites were mainly located in the ANK or TAD domain of NICD1, which are known to be crucial for the interaction between NICD1 and proteins, and transactivation activity, respectively. Individual mutation at T2063, T2090, or S2162 reduced NICD1 O-GlcNAcylation in response to OGT, PUGNAC, and GlcNAc, indicating their significance (Figure 4I and Supplemental Figure 5, C–E). Notably, pro–O-GlcNAcylation treatment failed to induce O-GlcNAcylation of the combined T2063A/T2090A/S2162A mutant NICD1, which was therefore used for subsequent functional analyses (Figure 4, J and K). Interestingly, the mutant NICD1 failed to undergo O-GlcNAcylation–induced nuclear accumulation, suggesting its role in nuclear translocation (Figure 4K and Supplemental Figure 5, F and G).

Analysis of the predicted crystal structure of NICD1 revealed that T2063 and T2090 were located in the ANK domain, whereas S2162 was located in the TAD domain (Supplemental Figure 5H). OGT overexpression failed to prevent the T2063A/T2090A/S2162A mutant NICD1 from binding to NUMB and decreased its ubiquitination, similar to WT NICD1 (Supplemental Figure 5I and Figure 4L). The findings that the affinity of T2063A/T2090A/S2162A mutant NICD1 to RBP-Jκ and DNA couldn’t be induced by PUGNAC and GlcNAc might be due to the O-GlcNAcylation site S2162 in the TAD domain (Figure 4, M and N, and Supplemental Figure 5J). Moreover, cells expressing the T2063A/T2090A/S2162A mutant NICD1 lacked OGT- or pro O-GlcNAcylation treatment–mediated effects on stress fiber remodeling, focal adhesion formation, acceleration of malignant properties, resistance to high stiffness–induced apoptosis, tumorigenesis, and resistance to cisplatin in cells with endogenous NICD1 knockout (Figure 4, O and P, Supplemental Figure 5, K–O, and Supplemental Table 6). Therefore, O-GlcNAcylation at T2063, T2090, and S2162 of NICD1 plays a crucial role in enabling chordoma cells to evade growth inhibition and relapse.

### Activation of LYN in response to stiff ECM mediates NICD1 O-GlcNAcylation by facilitating OGT catalytic activity.

**Figure 5:**
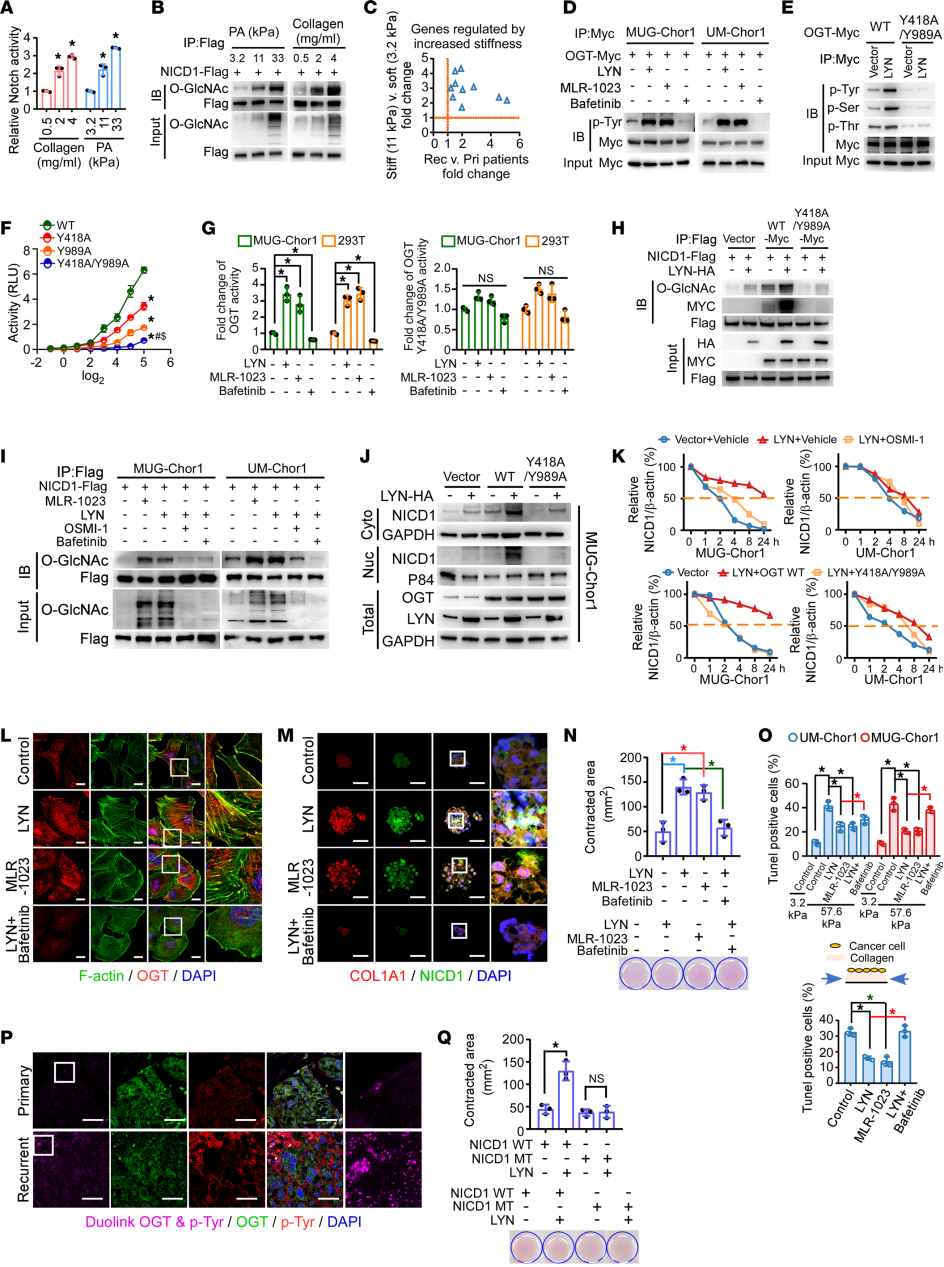
Activation of LYN in response to stiff ECM mediates NICD1 O-GlcNAcylation by facilitating OGT catalytic activity. (**A**) Dual luciferase reporter to assess Notch signaling activity in MUG-Chor1 cells cultured on substrates with different stiffnesses. (**B**) IP determined the effect of increased stiffness on NICD1 O-GlcNAcylation. (**C**) Expression of genes regulated by increased stiffness was assessed by qRT-PCR in primary and recurrent chordoma tissues, including HES1, HES5, HEY1, HEY2, NRARP, MYC, NANOG, OCT4, VCL, and SOX2. The orange dotted line indicates a fold change of 1. (**D**) IP was used to determine tyrosine phosphorylation of OGT. MLR-1023 (10 μM, 24 hours) was used to activate LYN tyrosine kinase, while bafetinib (10 nM, 24 hours) was utilized to inhibit LYN tyrosine kinase activity. (**E**) IP was used to identify tyrosine, serine, and threonine phosphorylation of WT and Y418A/Y989A OGT. (**F** and **G**) UDP-Glo Glycosyltransferase Assay (Promega) was used to detect glycosyltransferase enzyme activity of WT and mutant OGT. In **F**, symbols indicate significant differences versus specified groups: *WT; ^#^Y418A; ^$^Y989A. (**H** and **I**) IP was used to assess NICD1 O-GlcNAcylation in cells ectopically expressing WT or mutant OGT with or without LYN overexpression. (**J**) Effects of WT or mutant OGT with or without LYN overexpression on nuclear and cytoplasmic location of NICD1 protein were determined. (**K**) Whether O-GlcNAcylation inhibitor could abrogate effects of LYN overexpression on half-lives of NICD1 and whether mutation of OGT Y418/Y989 could cancel effects of WT OGT and LYN overexpression on half-lives of NICD1 were both assessed using WB. Quantification of Supplemental Figure 7, K and L. (**L**–**N**) Effects of LYN overexpression, kinase activation, or kinase activity suppression on F-actin, VCL, focal adhesions (**L**); COL1A1 (**M**); and cellular contractility (**N**) were assessed. Scale bar: 10 μm (**L**); 50 μm (**M**). (**O**) Effects of LYN overexpression, kinase activation, or kinase activity suppression on resistance to high stiffness– or stress-induced cell death were determined. (**P**) Duolink PLA to detect OGT-specific tyrosine phosphorylation in paired primary and recurrent chordoma tissues. Scale bar: 50 μm. (**Q**) In chordoma cells with NICD1 knockout, the collagen cell contraction assay was performed to assess effects of WT or T2063A/T2090A/S2162A (MT) NICD1 with or without LYN overexpression on MUG-Chor1 cellular contractility. Data in **A**, **F**, **G**, **N**, **O**, and **Q** are presented as mean ± SD. Statistical analysis was performed using 1-way ANOVA (**A**, **F**, **G**, **N**, **O**, and **Q**). *P < 0.05.

The detailed mechanism underlying the upregulation of NICD1 O-GlcNAcylation in recurrent tumors was further investigated. Accordingly, PA or type I collagen gels were used to simulate different stiffness levels of the substrates. Remarkably, as shown in Figure 5, A and B, and Supplemental Figure 6, A–D, elevated global and NICD1-specific O-GlcNAcylation, Notch activation, and expression of mechanical and CSC-related genes were observed in chordoma cells embedded on substrates with increased stiffness. Furthermore, genes associated with mechanical, CSCs, or Notch signaling that were upregulated by the stiff gel also exhibited higher expression in patients with recurrent chordomas than in patients with primary chordomas (Figure 5C and Supplemental Figure 6E).

We detected an interaction between OGT and LYN, a tyrosine kinase (Supplemental Figure 6, F and G). Interestingly, the phosphorylation of LYN Y397 and Y507, critical residues regulating LYN kinase activity, was enhanced when cultured on stiffer substrates, accompanied by Notch signaling activation (Supplemental Figure 6, H and I). Accumulated nuclear and cytoplasmic NICD1 in cells with ectopic expression of LYN or treatment with MLR-1023, a LYN kinase activator, and decreased NICD1 in response to bafetinib, an inhibitor of LYN kinase activity, were observed (Supplemental Figure 6J). The interaction between OGT and NICD1 was accelerated by LYN overexpression, as shown in Supplemental Figure 6, K and L. Increased NICD1-specific O-GlcNAc was observed in both the cytoplasm and nucleus, indicating that LYN kinase participates in increased stiffness-induced NICD1-specific O-GlcNAcylation (Supplemental Figure 6M).

No alteration in OGT abundance or location was observed. Increased tyrosine phosphorylation of OGT occurred in chordoma cells, particularly with LYN overexpression or MLR-1023 treatment, whereas bafetinib decreased OGT tyrosine phosphorylation (Figure 5D). Notably, phosphorylation of OGT at S4, S391, Y418, T419, T985, and Y989 was observed in response to LYN overexpression (Supplemental Figure 7A). A marked increase in OGT tyrosine phosphorylation in response to LYN overexpression, observed in WT OGT, was not observed in OGT carrying a mutation in Y418 or Y989 (Supplemental Figure 7B). As shown in Figure 5E, ectopic expression of LYN hardly induced tyrosine phosphorylation of OGT when combined with mutations inY418 and Y989. In vitro kinase assay further validated that Y418/Y989 of OGT was a substrate of LYN tyrosine kinase (Supplemental Figure 7C). The roles of tyrosine and serine phosphorylation in the regulation of OGT catalytic activity and translocation have been reported previously (34–37). No alteration in serine and threonine phosphorylation of OGT Y418A/Y989A suggested the modulation between nearby tyrosine, serine, and threonine residues.

As shown in Figure 5F, phosphorylation of OGT by LYN regulated its glycosyltransferase activity, as OGT Y989A and Y418A/Y989A mutants showed significantly reduced activity compared with WT OGT, indicating what we believe to be a novel mechanism regulating OGT catalytic activity in an LYN kinase-dependent manner (Figure 5G). Furthermore, LYN overexpression failed to promote the interaction between mutant OGT and NICD1, or enhanced NICD1 O-GlcNAcylation in cells expressing OGT mutants, suggesting a dependence on OGT activity (Figure 5H and Supplemental Figure 7D). Additionally, both LYN ectopic expression and increased kinase activity induced NICD1 O-GlcNAcylation and Notch signaling activation, which were abrogated by OSMI-1 (Figure 5I and Supplemental Figure 7, E–H). However, LYN failed to prevent NICD1 ubiquitination and degradation, prolong NICD1 half-life, or mediate the accumulation of cytoplasmic and nuclear NICD1 in cells expressing OGT Y418A/Y989A as it did in cells expressing WT OGT (Figure 5, J and K, and Supplemental Figure 7, I–L). In addition, there was no obvious alteration in the interaction between the NICD1 and the *HES1* promoters in response to LYN ectopic expression or activation in cells expressing OGT Y418A/Y989A (Supplemental Figure 7M).

Chordoma cells expressing LYN or incubated with MLR-1023 exhibited increased cellular contractility, focal adhesions, VCL, stress fibers, COL1A1 and FN1 protein levels, resistance to high stiffness or stress-induced death, and upregulated stemness, which was reversed by bafetinib or OSMI (Figure 5, L–O, and Supplemental Figure 8, A–H). As shown in Supplemental Figure 8I, bafetinib treatment markedly inhibited tumor growth when compared with the control group. We also observed relatively higher COL1A1 in clinical tumors expressing higher levels of LYN (Supplemental Figure 8J). Clinical validation showed increased OGT tyrosine phosphorylation in recurrent chordomas (Figure 5P), with a positive correlation between the OGT tyrosine phosphorylation level and NICD1 O-GlcNAcylation, Notch activity, and malignancy. Conversely, OGT tyrosine phosphorylation levels negatively correlated with RUNX2 expression (Supplemental Figure 8K).

Moreover, LYN overexpression promoted cellular contractility and interaction between RBP-Jκ and the *COL1A1* promoter in cells expressing WT NICD1, which was absent in cells expressing T2063A/T2090A/S2162A mutant NICD1 (Figure 5Q and Supplemental Figure 8L). These results indicate a critical role for the LYN/OGT/Notch1 axis in regulating mechanical remodeling in the perception of ECM stiffness.

### EPHA2 mediates LYN phosphorylation in response to ECM stiffness.

**Figure 6:**
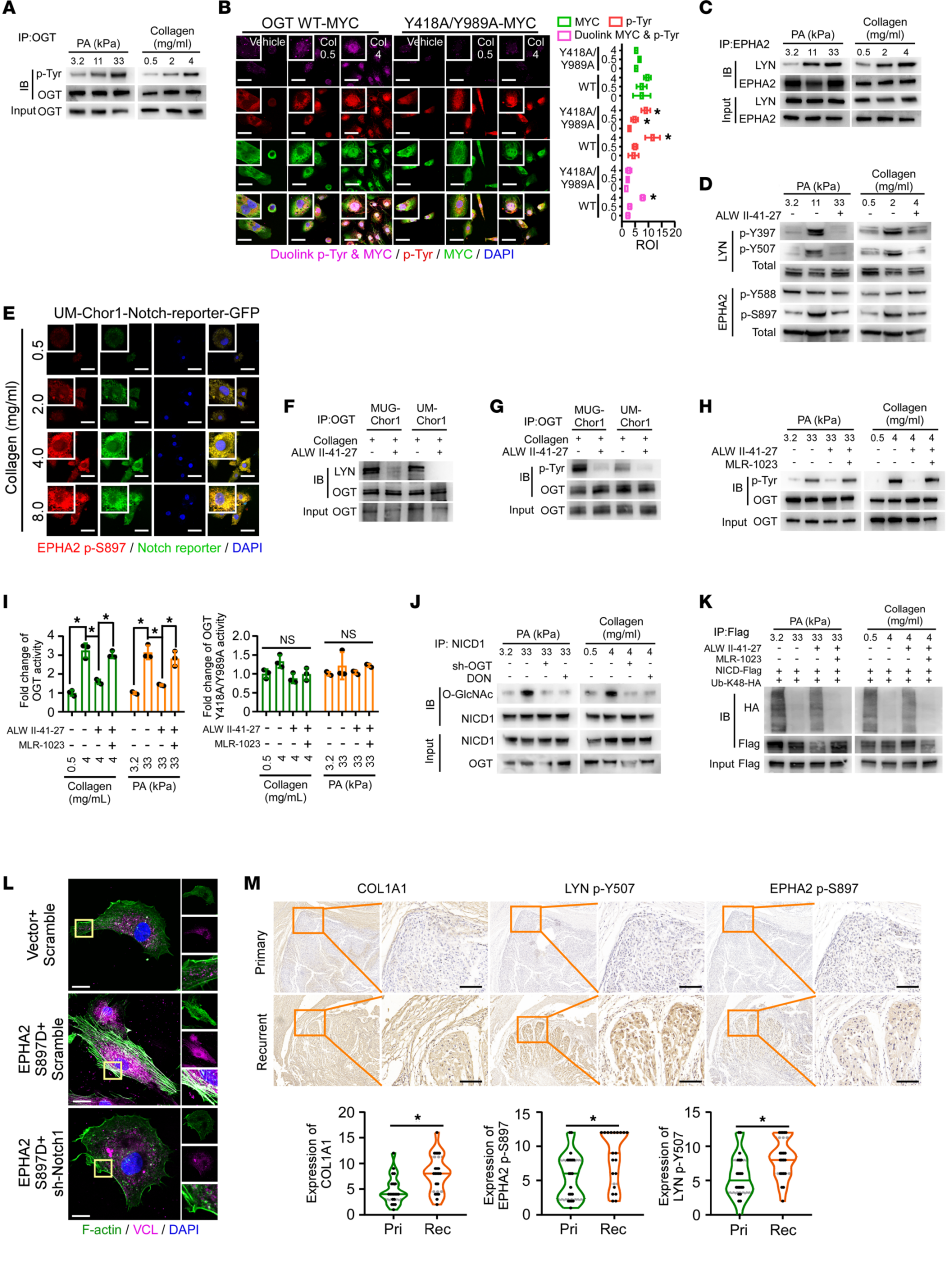
EPHA2 perceives ECM stiffness to induce LYN phosphorylation. (**A** and **B**) IP, Duolink PLA, and IF to identify OGT tyrosine phosphorylation, global tyrosine phosphorylation, and LYN phosphorylation in MUG-Chor1 cells cultured on substrates with different stiffnesses. Col is for type I collagen. Scale bar: 50 μm. (**C**) IP was used to identify the interaction between LYN and EPHA2 in MUG-Chor1 cells cultured on substrates with different stiffnesses. (**D**) The effects of EHPA2 inhibitor ALW II-41-27 (10 nM) on phosphorylation of LYN and EPHA2 in MUG-Chor1 cells cultured on substrates with different stiffnesses were assessed. (**E**) Phosphorylation of EPHA2 S897 along with Notch signaling activity in response to different stiffnesses were determined by IF. Scale bar: 50 μm. (**F** and **G**) IP was used to determine effects of high stiffness with EHPA2 inhibitor on the interaction between OGT and LYN and OGT tyrosine phosphorylation. Type I collagen (4 mg/mL). (**H**) IP was used to assess the effect of LYN kinase suppression on ALW II-41-27–mediated OGT tyrosine phosphorylation inhibition. (**I**) Glycosyltransferase activity of WT or mutated OGT was determined using the UDP-Glo Glycosyltransferase Assay. (**J**) The effects of silencing OGT or treatment of DON on NICD1 O-GlcNAcylation in MUG-Chor1 cells cultured on substrates with different stiffnesses were assessed. (**K**) The effects of MLR-1023 and ALW II-41-27 on NICD1 ubiquitination in cells cultured on substrates with different stiffnesses were determined by IP. (**L**) The effects of sustained EPHA2 S897 phosphorylation on F-actin, VCL, and focal adhesions were determined in the presence or absence of Notch1 silencing. Scale bar: 10 μm. (**M**) IHC was used to determine expression of COL1A1, EPHA2 p-S897, and LYN p-Y507 in primary and recurrent clinical chordoma tissues (*n* = 20 pairs). Scale bar: 100 μm. Data in **B** and **I** are presented as mean ± SD. Data in **M** are presented as mean (minimum to maximum). Statistical analysis was performed using paired Student’s *t* test (**M**) and 2-way ANOVA (**B** and **I**). *P < 0.05.

We further observed that OGT tyrosine phosphorylation increased in response to stiffer substrates; however, this effect was absent in cells in which OGT Y418 and Y989 residues were mutated simultaneously (Figure 6, A and B, and Supplemental Figure 9A). Interestingly, the stiff substrate facilitated the binding of Y507-phosphorylated LYN to WT OGT but not to the Y418A/Y989A mutant OGT (Supplemental Figure 9B). Additionally, we noticed that EPHA2, a receptor that responds to mechanical signals to trigger noncanonical signaling via phosphorylation at S897, could bind to LYN upon stimulation with increased stiffness (Figure 6C) (38). Furthermore, stiffer substrates induced the phosphorylation of EPHA2 at S897 and the activation of LYN, both of which were abrogated by ALW II-41-27, an EPHA2 inhibitor (Figure 6D). In parallel, as shown in Figure 6E and Supplemental Figure 9C, a positive correlation was observed between EPHA2 p-S897 and Notch signaling activity. Conversely, Y588, a tyrosine residue known to be phosphorylated upon ligand binding and inhibition of malignancy, remained unphosphorylated in response to a stiff ECM (Supplemental Figure 9D).

We further addressed the role of EPHA2 in ECM-mediated activation of Notch signaling. Inhibition of EPHA2 abrogated the interaction between LYN and OGT as well as OGT tyrosine phosphorylation induced by increased stiffness (Figure 6, F and G). Interestingly, the suppressive effects of ALW II-41-27 on OGT tyrosine phosphorylation were reversed by MLR-1023, indicating that EPHA2 regulates OGT in a LYN kinase-dependent manner (Figure 6H). Moreover, the effects of increased stiffness, ALW II-41-27, and MLR-1023 on the catalytic activity of WT OGT were not observed in the Y418A/Y989A mutant OGT (Figure 6I). The upregulated NICD1 O-GlcNAcylation, NICD1 nuclear translocation, and Notch signaling activity in stimulation of increased stiffness were remarkably repressed by silencing OGT or LYN or by treatment with DON (Figure 6J and Supplemental Figure 9, E–G). Furthermore, inhibition of NICD1 ubiquitination upon enhanced stiffness was reversed by ALW II-41-27, whereas MLR-1023 counteracted the ALW II-41-27–mediated promotion of NICD1 ubiquitination and degradation (Figure 6K). To further validate the EPHA2/Notch signaling pathway in downstream mechanical properties regulation, we constructed EPHA2 with continuous phosphorylation of S897 (EPHA2 S897D). As shown in Figure 6L and Supplemental Figure 9H, focal adhesions, VCL, stress fibers, Notch downstream genes, stemness-related genes, and mechanically related genes increased by EPHA2 activation could be repressed by silencing Notch1. Silencing LYN or inhibiting LYN kinase activity could abrogate EPHA2 S897D–induced phosphorylation of OGT tyrosine (Supplemental Figure 9I). Clinically and in vivo, we observed relatively higher levels of COL1A1, EPHA2 p-S897, and LYN p-Y507 in recurrent chordomas and subcutaneous tumors generated from MUG-Chor1-rec cells (Figure 6M and Supplemental Figure 9J). Taken together, our data suggested that ECM stiffness acts as a mechanical signal, inducing OGT catalytic activity to mediate NICD1 O-GlcNAcylation by triggering EPHA2-LYN signaling.

### Deletion of MIR31 contributes to LYN upregulation in recurrent chordoma.

**Figure 7:**
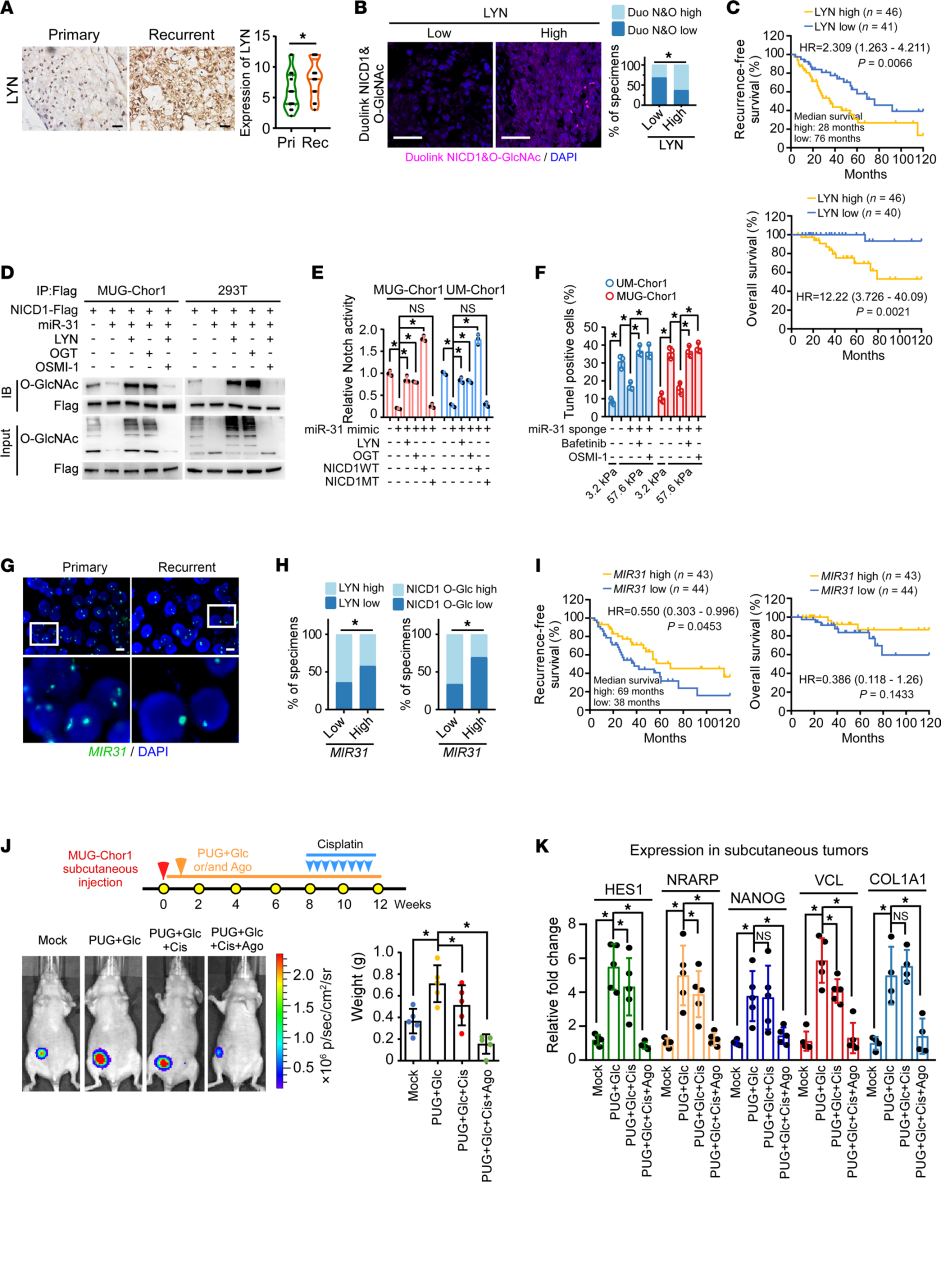
Deletion of *MIR31* contributes to LYN upregulation in recurrent chordoma. (**A**) IHC was used to analyze LYN expression in primary and recurrent chordoma tissues in clinic (*n* = 20 pairs). The solid line represents the mean value. (**B**) Duolink PLA was used to determine the level of NICD1 O-GlcNAcylation in chordoma. LYN expression was assessed using IHC. The median values of LYN and NICD1 O-GlcNAcylation were used as the cut-off values. *N* = 87. Scale bar: 50 μm. (**C**) Kaplan-Meier analysis of recurrence-free survival and overall survival. Patients with chordoma were grouped according to the LYN level detected by IHC. Median survival time and horizon risk (HR) of recurrence and death are listed. (**D**) IP was used to determine the effects of LYN, OGT, and OSMI-1 on miR-31 mimic-regulated NICD1 O-GlcNAcylation. (**E**) Dual luciferase assay was used to assess the effects of LYN, OGT, WT NICD1, and T2063A/T2090A/S2162A (MT) NICD1 on Notch signaling activity. (**F**) TUNEL assay results from cells cultured on substrates with different stiffnesses and indicated treatments. (**G**) Fluorescence in situ hybridization was used to determine levels of genomic *MIR31* in paired primary and recurrent chordoma tissues. Scale bar: 10 μm. (**H**) Patients with chordoma grouped by level of *MIR31* determined by qRT-PCR (*n* = 87). LYN protein expression was determined by IHC. NICD1 O-GlcNAcylation level was determined by Duolink PLA. (**I**) Kaplan-Meier analysis of recurrence-free survival and overall survival. Patients with chordoma were grouped according to the *MIR31* DNA level detected by qRT-PCR. Median survival time and HR of recurrence and death are listed. (**J** and **K**) 10^7^ MUG-Chor1 cells were inoculated subcutaneously. Tumors were treated with PUGNAC (50 μM), GlcNAc (10 μM), or miR-31 agomir (1 mg/mL). After 6 weeks, cisplatin (cis-diamminedichloroplatinum, 4 mg/kg) was intraperitoneally injected every 3 days (*n* = 5/group). (**J**) Representative bioluminescent images of subcutaneous tumors with indicated treatment at 12 weeks are shown, and the tumor weight is presented. (**K**) Expression of Notch downstream genes and CSC- and mechanical-related genes was assessed using qRT-PCR. Data in **A** are presented as mean (minimum to maximum). Data in **E**, **F**, **J**, and **K** are presented as mean ± SD. Statistical analysis was performed using paired Student’s *t* test (**A**), χ^2^ test (**B** and **H**), and 1-way ANOVA (**E**, **F**, **J**, and **K**). *P < 0.05.

LYN protein levels were upregulated in recurrent chordoma and positively correlated with NICD1-specific O-GlcNAcylation (Figure 7, A and B). Patients with chordoma obtaining higher LYN expression exhibited shorter recurrence-free survival time, shorter overall survival time, and a significantly increased risk of recurrence compared with those with lower LYN expression (HR = 2.309, 95% CI, 1.263–4.211) (Figure 7C). However, the mechanisms underlying LYN upregulation require further investigation. We observed that miR-31 could target the 3′UTR of LYN mRNA and downregulate LYN expression (Supplemental Figure 10, A and B). Moreover, overexpression of LYN or OGT abrogated the miR-31–induced decrease in NICD1 O-GlcNAcylation and Notch signaling, as well as the inhibition of malignancy, which could be counteracted by OSMI-1. Notably, the expression of WT NICD1, but not mutant NICD1, also reversed the miR-31–induced inhibition of Notch signaling (Figure 7, D and E, and Supplemental Figure 10, C and D). Chordoma cell lines stably expressing miR-31 sponge were utilized. The miR-31 inhibition increased expression of stemness-related genes, promoted generation of numerous large spheres, and mediated resistance to high stiffness–induced cell death, which could be abrogated by inhibiting LYN or OGT (Figure 7F and Supplemental Figure 10, E and F). In support of our hypothesis that increased LYN was attributable to the downregulation of miR-31, the *MIR31* genomic copy number was relatively lower in recurrent chordomas than in primary tumors (Figure 7G and Supplemental Figure 10G). This finding was supported by the negative correlation between *MIR31* DNA and LYN protein levels in clinical samples (Figure 7H). The negative correlation between *MIR31* and NICD1 O-GlcNAcylation levels was also determined (Figure 7H). Patients with chordoma-bearing tumors with lower *MIR31* DNA level had shorter recurrence-free survival and overall survival times (Figure 7I). Besides, patients with low *MIR31* had a median recurrence-free survival of 38 months, whereas those with high *MIR31* reached 69 months. In our cohort, patients with higher *MIR31* exhibited a significantly decreased risk of recurrence compared with those with lower *MIR31* (HR = 0.55; 95% CI, 0.303–0.996), suggesting that *MIR31* deletion may serve as a prognostic marker for recurrence outcomes. To validate the tumor-suppressive role of miR-31 in vivo, an miR-31 agomir was used. As shown in Figure 7J, the increased tumorigenesis capacity induced by pro–O-GlcNAcylation treatment could only be partly abrogated by cisplatin, but the miR-31 agomir increased the sensitivity of chordoma cells to cisplatin. Moreover, the combination of cisplatin and the miR-31 agomir remarkably suppressed the growth of subcutaneous tumors. Subcutaneous tumors treated with a combination of the miR-31 agomir and cisplatin exhibited lower expression of Notch signaling activity, CSC-like properties, or mechanical property–related genes than those treated with PUGNAC, GlcNAc, or cisplatin alone (Figure 7K). Therefore, we speculated that chordoma cells with upregulated LYN due to deletion of *MIR31* tended to sense increased ECM stiffness, subsequently inducing remodeling of mechanical properties to adapt to the mechanical microenvironment.

## Discussion

Accumulating evidence has revealed the pivotal role of mechanical forces within the tumor microenvironment, including stress, stiffness, adhesiveness, and viscoelasticity, in driving tumor progression (39, 40). Abnormalities in ECM stiffness disrupt tissue homeostasis and contribute to cellular dysfunction (41). As previously reported, the increased rigidity of the NP induces iron death in NP cells during disc herniation (42, 43). Our findings demonstrate that a high-stiffness matrix inhibits proliferation and promotes programmed cell death in vitro. Interestingly, tumor cells exhibit remarkable mechanical adaptability, dynamically responding to ECM mechanical signals via ECM remodeling or cytoskeletal reconfiguration (14–18). Primary chordomas typically arise from remnants of primitive embryo notochord tissue, which display soft jelly-like properties (<2–4 kPa), whereas recurrent chordomas originate from infiltrating tumor cells facing a stiffer microenvironment, including bones (usually >50 kPa) (10–13). The difference in the mechanical properties of the microenvironment indicates that tumor cells infiltrating bones require dynamic mechanical adjustment to survive. Impressively, we found that recurrent chordoma cells as well as cell lines surviving high stiffness displayed increased stress fiber formation, focal adhesion assembly, and proteoglycan accumulation. Notch1 O-GlcNAcylation mediates mechanical adaptation and resistance to high stiffness– or stress-mediated cell death, which can be markedly abrogated by an actin polymerization inhibitor, suggesting potential therapeutic targets. Interestingly, in some patients, the primary lesion is typically characterized by a mucus-rich stringy tissue, whereas recurrent lesions are cellular, poorly differentiated, or even dedifferentiated, suggesting increased tumor stemness. In conclusion, we propose a model of chordoma recurrence in which cells match their stiffness with the substrate through cytoskeletal modulation and possibly strain stiffening and secret more collagen and proteoglycan into ECM, subsequently promoting survival against high stress. Enhanced tumor stemness facilitates recurrent tumor development in a small number of surviving cells. However, more rigorous quantitative standards would improve the quantitative assessment of mechanoadaptive processes in future, such as single-cell mechanophenotyping and computational modeling. Moreover, the applicability of our findings to various chordoma subtypes warrants further investigation.

The detailed mechanism by which chordoma cells sense, transmit, and react to stiff ECM and modulate their function remains unclear. Our findings revealed that chordoma cells expressing higher levels of LYN exhibited an inclination to perceive ECM with elevated stiffness, consequently activating Notch signaling through the promotion of Notch1 O-GlcNAcylation, thus facilitating mechanical adaptation. EPHA2, a member of the ephrin receptor family and receptor tyrosine kinase predominantly located in the cell membrane, participates in extracellular signal transduction. Similar to other members of the ephrin receptor family, EPHA2 comprises an extracellular segment containing ligand-binding domains, a single transmembrane domain, and an intracellular segment containing tyrosine kinase domains. Under normal physiological conditions, EPHA2 can be activated by ligands, such as Ephrin A2, which plays pivotal roles in nervous system development, axonal guidance, synaptic function, cell migration, adhesion, and vascular remodeling (44). In the present study, we revealed EPHA2’s role as a mechanosensor, capable of ligand-independent activation by directly phosphorylating the tyrosine kinase, LYN. Subsequently, LYN phosphorylation triggered OGT-mediated Notch1 O-GlcNAcylation. Therefore, we identified what we believe to be a novel mechanical and biological signal transduction pathway that ultimately mediates adaptation to the mechanical microenvironment.

Under normal physiological conditions, Notch signaling is tightly regulated by negative-feedback mechanisms (25). For example, NUMB facilitates the degradation of NICD1 in the cytoplasm, thereby impeding its nuclear translocation (45–47). Within the nucleus, the CDK8-FBXW7 complex targets NICD1 for ubiquitin-dependent degradation upon transcription initiation, thus curbing the activation of Notch signaling (48–50). Understanding how negative regulation is disrupted to sustain high levels of Notch signaling in recurrent chordomas warrants further investigation. In other tumor contexts, such as pancreatic ductal adenocarcinoma, the extracellular EGF-like repeats of the extracellular domain are O-GlcNAcylated by EOGT, enhancing ligand binding and promoting Notch1 cleavage; however, the effect of EOGT-mediated O-GlcNAcylation of the extracellular domain on the released NICD1 remains unclear (27, 30). Our study revealed what we believe to be a novel finding: that the intracellular domain of Notch1 can also undergo O-GlcNAcylation. We observed increased global O-GlcNAcylation in recurrent chordomas accompanied by overactivated Notch signaling and identified T2063, T2090, and S2162 as potentially novel O-GlcNAcylation sites. Interestingly, we noticed that O-GlcNAcylation of T2063 and T2090, which are crucial for protein interactions within the ANK domain, may prevent NICD1 from binding to NUMB, explaining the observed increase in NICD1 stability in the cytoplasm. Furthermore, O-GlcNAcylation promotes NICD1 binding to the promoters of *HES1* and specific aggressiveness-related genes, enhancing transcriptional activity, likely mediated by S2162, located in the TAD domain involved in DNA interaction. Thus, we have identified a posttranslational modification of NICD1 that regulates Notch activation during chordoma recurrence. Further exploration is warranted to elucidate whether the altered DNA-binding affinity and NUMB interactions result from O-GlcNAcylation–induced conformational changes. While our experimental models demonstrate a functional role of Notch1 in recurrence pathways, its clinical causality requires further validation through prospective studies.

OGT and OGA enzymes govern O-GlcNAc cycling in response to nutrients and stress, with disruptions implicated in various human diseases (51). To investigate whether and how the dynamic cycling of OGT-OGA is disrupted in recurrent chordomas, we identified two tyrosine residues, Y418 and Y989, whose phosphorylation regulates OGT catalytic activity. Specifically, Y989, which is situated within the C-terminal catalytic domain, plays a crucial role in the regulation of OGT activity and is likely phosphorylated by LYN kinase. Y418, on the other hand, is located within the TPR repeats, suggesting its importance in protein interactions. Thus, we established what we believe to be the novel effects of LYN kinase on OGT substrate selectivity and catalytic activity. *MIR31* deletion-mediated LYN upregulation appears to lead to addition of O-GlcNAc to NICD1, with potential implications for therapeutic strategies targeting Notch or OGT in chordoma. Notably, miR-31 agomir enhanced chordoma sensitivity to chemotherapy by suppressing Notch signaling and tumor stemness. Further investigations are warranted to validate the clinical significance of *MIR31* deletion as a predictive biomarker for chordoma recurrence owing to a small sample size in this study. While miR-31 agomir shows therapeutic potential, its clinical applicability and safety need further evaluation. Future research should also focus on exploring the detailed therapeutic strategies from which the patients with *MIR-31* deletion could benefit.

## Methods

### Sex as a biological variable.

Sex was not considered as a biological variable in the design and reporting of this study. Human chordoma samples were obtained from 61 male and 26 female patients. For in vivo experiments, immunodeficient mice of both sexes were used for xenograft assays. However, formal analyses of sex-specific effects were not performed, and potential sex-related influence on tumor behavior cannot be excluded. Therefore, while the findings are expected to be broadly relevant to both sexes, this limitation has been acknowledged.

### Cell lines.

All chordoma cell lines were obtained from the ATCC and cultured according to the manufacturer’s instructions. Human primary NP cells were obtained from ScienCell and cultured in the NP cell medium (ScienCell). The authenticity of the cell lines was verified by short tandem repeat fingerprinting at Guangzhou Cellcook Biotech Co. Ltd. MUG-Chor1 and UM-Chor1 cells were cultured in medium containing Iscove’s Modified Dulbecco’s medium: RPMI-1640 medium (4:1), 10% FBS, and additional 1% L-glutamine. JHC7 cells were cultured in medium containing DMEM: F12 and 10% FBS. HEK293T (293T) cells were obtained from the Cell Bank of Shanghai Institutes of Biological Sciences and cultured in DMEM (GIBCO) medium supplemented with 10% FBS and 1% penicillin/streptomycin (penicillin 100 U/mL and streptomycin 10 μg/mL).

### Human chordoma tissues.

The study comprised 87 consecutive cases of surgically treated primary chordoma at The First Affiliated Hospital of Sun Yat-sen University and Sun Yat-sen Memorial Hospital of Sun Yat-sen University between January 2000 and June 2017, with complete FFPE specimen collection from all primary tumors and additional matched recurrence specimens, which were available for 24 cases. All 87 patients were ethnically Chinese. Following histological review and quality control, 6 matched pairs were processed for mRNA microarray analysis, 10 pairs for qPCR validation, and 20 pairs for IHC. The histological characterization and clinicopathological staging of the cases were determined following the standards provided in the current Union for International Cancer Control Tumor-Node-Metastasis classification. The clinicopathological characteristics of patients with chordoma are presented in Supplemental Tables 1 and 2.

### Duolink proximity ligation assay.

To determine NICD1 O-GlcNAcylation, OGT tyrosine phosphorylation, and the interaction between OGT and LYN, chordoma cells, spheroids, and tumor tissues were subjected to Duolink proximity ligation assay (PLA) in situ (DUO92101, Sigma-Aldrich) according to the manufacturer’s instructions. The samples were stained with anti–O-GlcNAc (ab2739, Abcam), anti-cleaved-Notch1 (4147, Cell Signaling Technology), anti-Flag (F7425, Sigma-Aldrich), anti-p-Tyr (ab10321, Abcam), anti-Myc (2272, Cell Signaling Technology), anti-OGT (ab177941, Abcam), anti-Myc (2276, Cell Signaling Technology), and anti-LYN p-Y507 (ab33914, Abcam) primary antibodies overnight at 4°C, followed by incubation with corresponding secondary antibodies conjugated with Duolink PLA Probes (DUO92001 and DUO92005, Sigma-Aldrich) for 1 hour at 37°C in the dark. Duolink In Situ Detection Reagents FarRed (DUO92013, Sigma-Aldrich) were utilized further. Nuclei were shown by DAPI. Images were captured using a confocal microscope (Leica SP8).

### Cell culture on gel substrates with different stiffness.

PA and collagen gels are commonly used in cell mechanics experiments. PA gels have highly adjustable mechanical properties and a wide range of Young’s moduli, while collagen gels are widely used in cell biology research. The details of PA gel preparation with calibrated stiffness are described in the Supplemental Methods, as described previously (52). Glass coverslips were coated with silane (Sigma-Aldrich). PA gels with variable rigidities were prepared by mixing different ratios of bis-acrylamide, acrylamide, and sulfo-SANPAH (Sigma-Aldrich). The gels were photoactivated using ultraviolet. Gels were crosslinked overnight with type I collagen, cells were seeded on top of the gels, and cells were cultured at 37°C. For RNA and protein analyses, cells were cultured for 48 hours and subsequently digested with 0.25% trypsin. The digested cells were washed with PBS and subjected to Western blot (WB), IP, or quantitative real-time PCR (qRT-PCR). For the colony formation assay, the cells were cultured for 7 days, and the colonies were stained with crystal violet. The number of clones was counted under each condition. For the TUNEL assay, cells were cultured for 48 hours, fixed in 4% formaldehyde (in PBS), and then followed by the manufacturer’s protocol (Abcam).

Details regarding collagen gel preparation are described in the Supplemental Methods and are according to a modified protocol from Artym and Matsumoto (53). Chordoma cells were embedded in collagen gel. After gel polymerization, cell culture medium was then added carefully on top of the cultures, and cells were cultured at 37°C. For RNA and protein analyses, the cells were cultured for 48 hours. The collagen embedded cultures were digested with collagenase (Sigma-Aldrich) for 10 minutes at 37°C. The digested products were washed with PBS. Cell pellets were subjected to WB, IP, or qRT-PCR. For immunofluorescence (IF) staining, cells were reseeded on a coverslip. After 6 hours, cells were fixed and subjected to Duolink PLA or IF analysis.

### Collagen cell contraction assay.

A Collagen-based Contraction Assay Kit (2-Step Attached Model; Cell biolabs) was utilized (40). The cells were harvested and resuspended in the desired medium at 2 × 10^6^ to 5 × 10^6^ cells/mL. The collagen lattice was prepared by mixing 2 parts of the cell suspension with 8 parts of a cold Collagen Gel Working Solution. 0.5 mL of the cell-collagen mixture was added per well in a 24-well plate and incubated for 1 hour at 37°C. After collagen polymerization, 1.0 mL culture medium was added to each collagen gel lattice, followed by incubation for 2 days, during which time stress developed. The cells were treated with 10 mM 2,3-butanedione 2-monoxime before releasing the stressed matrix. To initiate contraction, collagen gels were gently released from the sides of the culture dishes using a sterile spatula. Changes in the collagen gel size (contraction index) were measured using a ruler.

### Mechanical stretch of cultured cells.

We employed a uniaxial cell stretcher system to investigate cellular responses to mechanical forces by simulating mechanical stimuli. Chordoma cells were plated in 4-well culture plates precoated with type I collagen, which mimicked the extracellular matrix environment. The chordoma cells were seeded into the chambers and incubated overnight before cell stretching. The wells were cyclically stretched at a 10% compression rate (achieving a compression rate of 1 second, held for 1 second, and then returned to the initial state in 1 second) for a total of 3 hours using a CellTank System (CELL & FORCE, Hangzhou Surface Force Technology), with statically cultured wells as controls. The 10% stretch/compression magnitude was selected based on the capacity of the mechanical device to elongate or shorten the chamber by 10% of its original length, thereby applying a relevant tensile or compressive load to adherent cells (54, 55). The TUNEL assay was employed to assess cellular damage resulting from mechanical stress. Images were captured using a laser confocal microscopy.

### Xenograft analysis.

BALB/c nude mice (male and female, 4–5 weeks of age, 12–14 g) were obtained from Beijing Vital River Laboratory Animal Technology Co. Ltd. The mice were housed in specific pathogen–free facilities on a 12-hour-light/dark cycle at a temperature of 18°C–22°C and humidity of 50%–60%. The indicated chordoma cells (1 × 10^7^) resuspended in PBS and Matrigel (356234, BD) were subsequently injected, and the tumors were monitored every 2 weeks. Tumor length (*L*) and width (*W*) were measured weekly using a digital caliper, and tumor volumes (*V*) were calculated using the formula *V* = *L* × *W*^2^/2. At the indicated experimental endpoints, mice were anesthetized and sacrificed, and tumors were resected, sectioned (5 μm in thickness), and histologically examined by H&E staining. To construct a murine model of recurrent chordomas, 10^7^ cells were inoculated subcutaneously (*n* = 10). After 42 days, a large part of the tumor was resected and cultured; it was then used to generate the MUG-Chor1-pri cell line. A residual tumor of approximately 3 mm^2^ was left, which grew into a larger tumor after another 42 days; this tumor was then used to generate MUG-Chor1-rec cell line.

### Statistics.

Survival curves were established using the Kaplan-Meier method and compared using the log-rank test. For mRNA array data, genes were considered differentially expressed if they exhibited a |log_2_ fold change| ≥ 1 with an FDR-adjusted *P* < 0.05 (Benjamini-Hochberg method). Student’s *t* test (2-tailed) or the 2-tailed paired *t* test was used for comparisons between 2 groups or paired samples, respectively, following confirmation of normality by the Shapiro-Wilk test (*P* > 0.05). For comparisons among 3 or more groups, 1-way ANOVA followed by Tukey’s honestly significant difference post hoc test was used when assumptions of normality and homogeneity were met. Differences in the distribution of categorical variables were evaluated using Pearson’s χ^2^ test. All statistical analyses, except for the sequencing data, were performed using SPSS (IBM, Armonk) software package and GraphPad Prism 8 version 8.3.0 (GraphPad Software). Data are presented as mean ± SD, mean (95% CI), or mean (minimum to maximum), as indicated in the figure legends. In all cases, *P* < 0.05 was considered statistically significant.

### Study approval.

All animal experimental procedures were approved by the Institutional Animal Care and Use Committee of Sun Yat-sen University ([2018]-170) and Institutional Research Ethics Committees of Chongqing Medical University (K2023-198). Donors provided prior written informed consent, and approval from the Institutional Research Ethics Committees of Chongqing Medical University (K2023-198) and Sun Yat-sen University ([2018]-170) were obtained.

### Data availability.

All reported data values are available in the Supporting Data Values file. The raw data for ChIP-seq and mRNA microarray analyses are available in the Gene Expression Omnibus (GEO) database under accessions GSE313846 and GSE312699, respectively. Any additional information from this study is available from the corresponding author upon reasonable request.

## Author contributions

Conceptualization: LL and CL. Methodology: CL, WP, YY, JL, XS, YP, ZQ, and SW. Investigation: WP, YY, JL, XS, YP, ZQ, and SW. Clinical specimens and clinical data: CL, PS, DH, ZL, and XW. Supervision: LL and CL. Discussion of project ideas: LL, CL, and WP. Writing of the original draft of the manuscript: CL and WP. Review and editing of the manuscript: LL, CL, and WP. The order of the co–first authors was assigned based on their specific contributions and efforts to the study.

## Funding support

- National Natural Science Foundation of China (82473020).
- Natural Science Foundation of Chongqing Science and Technology Commission (CSTB2023NSCQ-LZX0018, CSTB2022NSCQ-MSX0929, CSTB2024NSCQ-MSX0952).
- Chongqing High-Level Medical Talent Program for Young and Middle-Aged Individuals (YXGD202415).
- Chongqing Science and Technology Bureau and Health Commission Joint Fund (2024GDRC006).
- The Science and Technology Research Project of Chongqing Education Commission (KJQN202200441).
- Joint Funds for the Innovation of Science and Technology, Fujian province (2025Y9281).
- Fujian Provincial Natural Science Foundation of China (2025J01127).
- Research project of Fujian Medical University Union Hospital (2024XH034).

## Supplementary Material

Supplemental data

Unedited blot and gel images

Supporting data values

## Version 1 02/03/2026

In-Press Preview

## Version 2 03/16/2026

Electronic publication
