# Supplementary material for: Notch1 O-GlcNAcylation drives tumor stemness and mechanoadaptation to a stiff microenvironment and promotes chordoma recurrence
Source: J Clin Invest. 2026 Feb 3;136(6):e194378. doi: 10.1172/JCI194378 (PMC12987655; doi:10.1172/JCI194378)
Supplement: Supplemental data [file jci-136-194378-s313.pdf]

Supplementary Information

Mechanoadaptation for high stiffness-resistance and tumor stemness, driven by Notch1 O-GlcNAcylation, promote chordoma recurrence

Lian, et al.

Supplemental Figure and Figure legends

Supplemental Figure 1

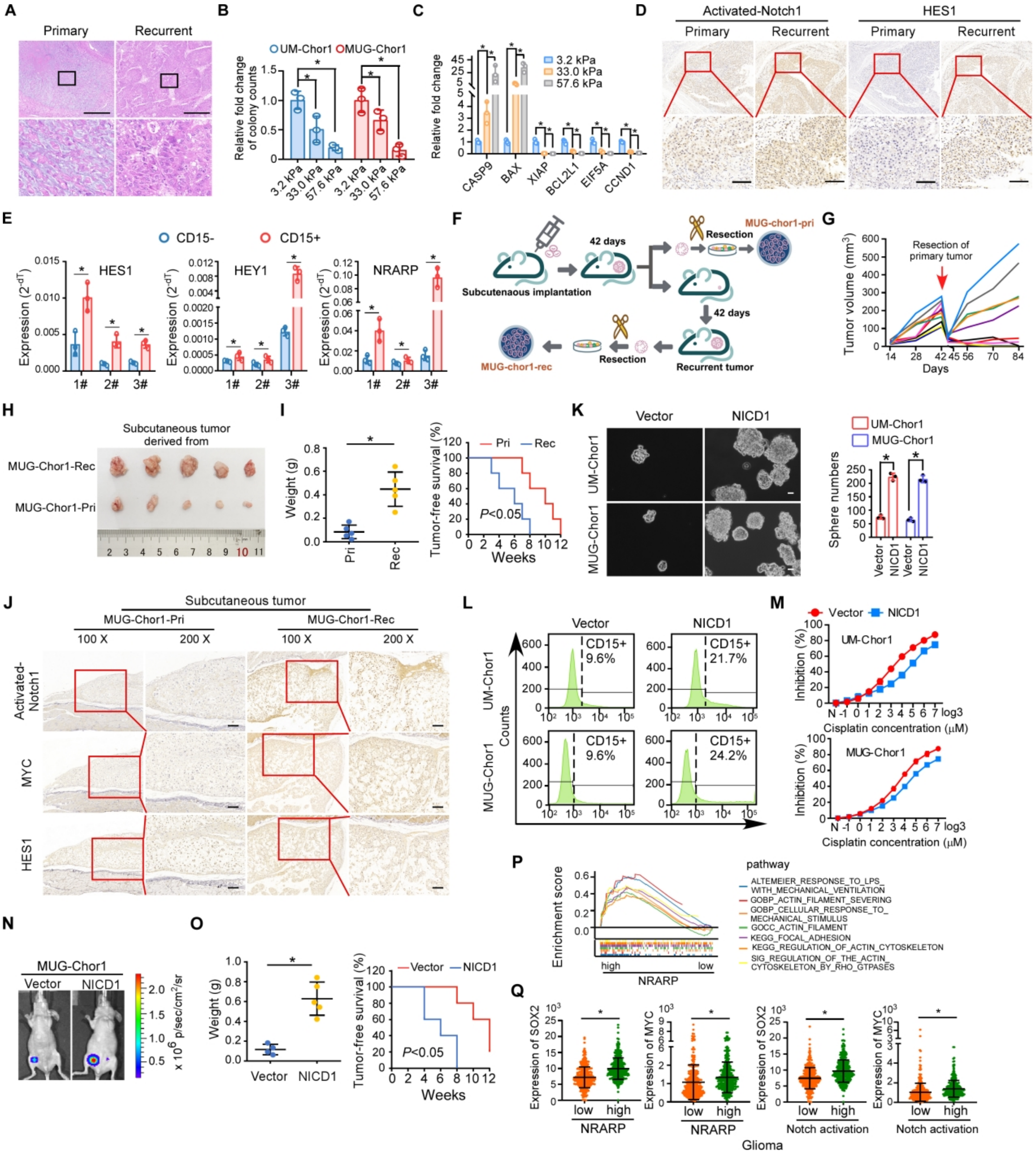

Supplemental Figure 1. Mechanical properties remodeling and increased tumor stemness were identified during chordoma recurrence. (A) H&E staining of chordoma tissues. Scale bar: 500 μm. **(B and C)** The effects of increased stiffness on colony-formation ability and expression of proliferation- or apoptosis-related genes were assessed. **(D)** IHC staining detects Notch activity in chordoma specimens. Scale bar: 100 μm. **(E)** qRT-PCR to assess Notch downstream genes in CD15+ or CD15-

chordoma tumors cells which were extracted by flow cytometry. **(F)** The construction of a recurrent chordoma mouse model (detail in the METHODS section, n = 10). **(G)** The growth curve of tumors from (F). **(H** and **I)** 10^7 chordoma cells were subcutaneously inoculated into the nude mice (n = 5 per group). All cases were presented at 12 w. The weight and tumor-free survival time were assessed (I). **(J)** IHC analysis to detect Notch activity and stemness-related gene MYC in the subcutaneous tumors from (H). Scale bar:100 μm. **(K)** Sphere formation was performed in chordoma cells with ectopic expression of NICD1. Scale bar: 50 μm. **(L)** The fraction of CD15 positive cells was assessed in chordoma cells with ectopic expression of NICD1. **(M)** IC50 assay to assess the cytotoxicity of cisplatin on chordoma cell lines when NICD1 was ectopically expressed. **(N** and **O)** 10^7 NICD1-overexpressing and corresponding MUG-Chor1-luci cells were subcutaneously inoculated into the nude mice (n = 5 per group). Representative bioluminescent images of subcutaneous tumors are shown (N). Weight of the tumors and the tumor-free survival time were determined (O). **(P)** GSEA analysis to determine the correlation between NRARP expression and mechanical properties-related signature in patients with gliomas from TCGA datasets. **(Q)** Expression of SOX2 and MYC levels of 699 patients with gliomas in TCGA datasets grouped according to NRARP and Notch signaling activity. Notch signaling activity was assessed by the expression of a combination of Notch signaling-related genes, HES1, HES5, HEY1, HEY2, and NRARP. Data in panels B, C, E, I, K, M, O, and Q are presented as mean ± SD. Statistical analysis was performed using unpaired Student's *t* test (E, I, K, O, and Q) and one-way ANOVA (B and C). *: $P < 0.05$.

Supplemental Figure 2

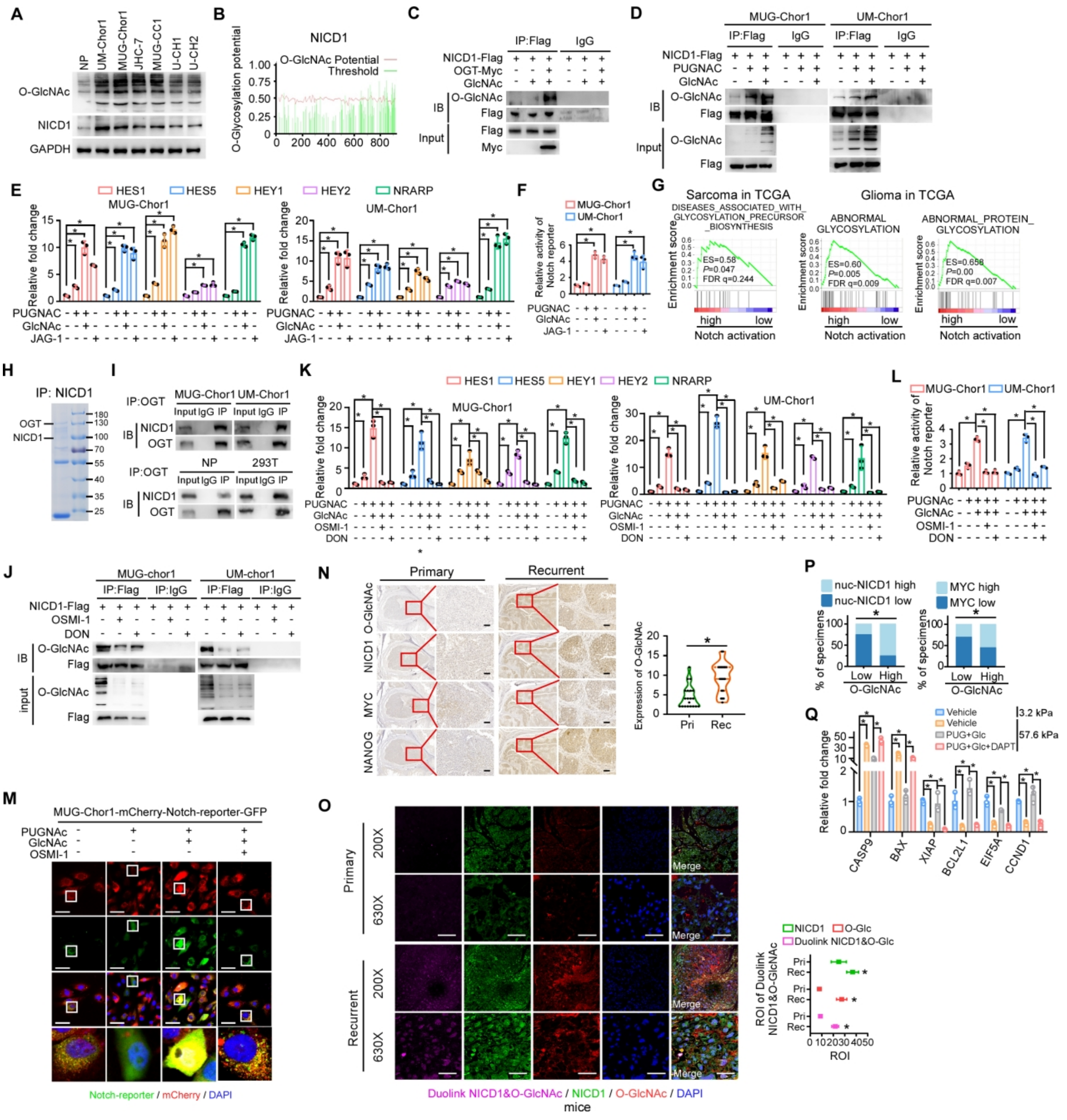

Supplemental Figure 2. Increased NICD1 O-GlcNAcylation contributes to Notch signaling aberrant activation in recurrent chordoma. (A) WB to detect NICD1 and O-GlcNAc in established NP and chordoma cell lines. **(B)** The potential O-GlcNAcylation sites on NICD1 predicted by NetOGlyc Server on line (https://www.dtu.dk/english). **(C)** IP to detect the effects of OGT and GlcNAc on NICD1 O-GlcNAcylation. **(D)** IP to analyze the effects of PUGNAC (50 μM) and GlcNAc (10 μM) on NICD1 O-GlcNAcylation. **(E)** Notch signaling downstream genes expression in chordoma cells in response to PUGNAC, GlcNAc, and JAG-1

was analyzed by qRT-PCR. **(F)** Dual luciferase reporter to determine Notch signaling activity. **(G)** GSEA analysis in sarcoma and glioma patients from TCGA dataset to determine the correlation between Notch activation and GlcNAcylation-related signature. **(H)** IP and MS to identify the interacting proteins of NICD1. **(I)** IP to determine endogenous interaction between OGT and NICD1 in chordoma cells, NP, and 293T cells. **(J)** IP to assess the effect of OSMI-1 (50 μM) and DON (100 μM) on NICD1 O-GlcNAcylation. **(K** and **L)** qRT-PCR and dual luciferase reporter to determine the effects of PUGNAC, GlcNAc, OSMI-1, and DON on Notch signaling activity. **(M)** IF to determine Notch signaling activation. Scale bar: 50 μm. **(N)** IHC assay detecting NICD1, O-GlcNAc, NANOG, and MYC in clinical chordoma specimens (n = 20 pairs). Scale bar:100 μm. **(O)** NICD1 specific O-GlcNAcylation in subcutaneous tumors developed from MUG-Chor1-pri and rec cells was identified by Duolink PLA and IF assay. Scale bar: 50 μm. **(P)** IHC to detect nuclear NICD1, O-GlcNAc, and MYC in clinical specimens (n = 40). Median value was used as the cut-off value. **(Q)** qRT-PCR to detect the effects of PUGNAC, GlcNAc, and DAPT on cell death in cells cultured on substrates with indicated stiffness. Data in panel N is presented as mean (min to max). Data in panels E, F, K, L, O, and Q are presented as mean ± SD. Statistical analysis was performed using paired Student's *t* test (N), unpaired Student's *t* test (O), Chi-square test (P), and one-way ANOVA (E, F, K, L, and Q). *: $P < 0.05$.

Supplemental Figure 3

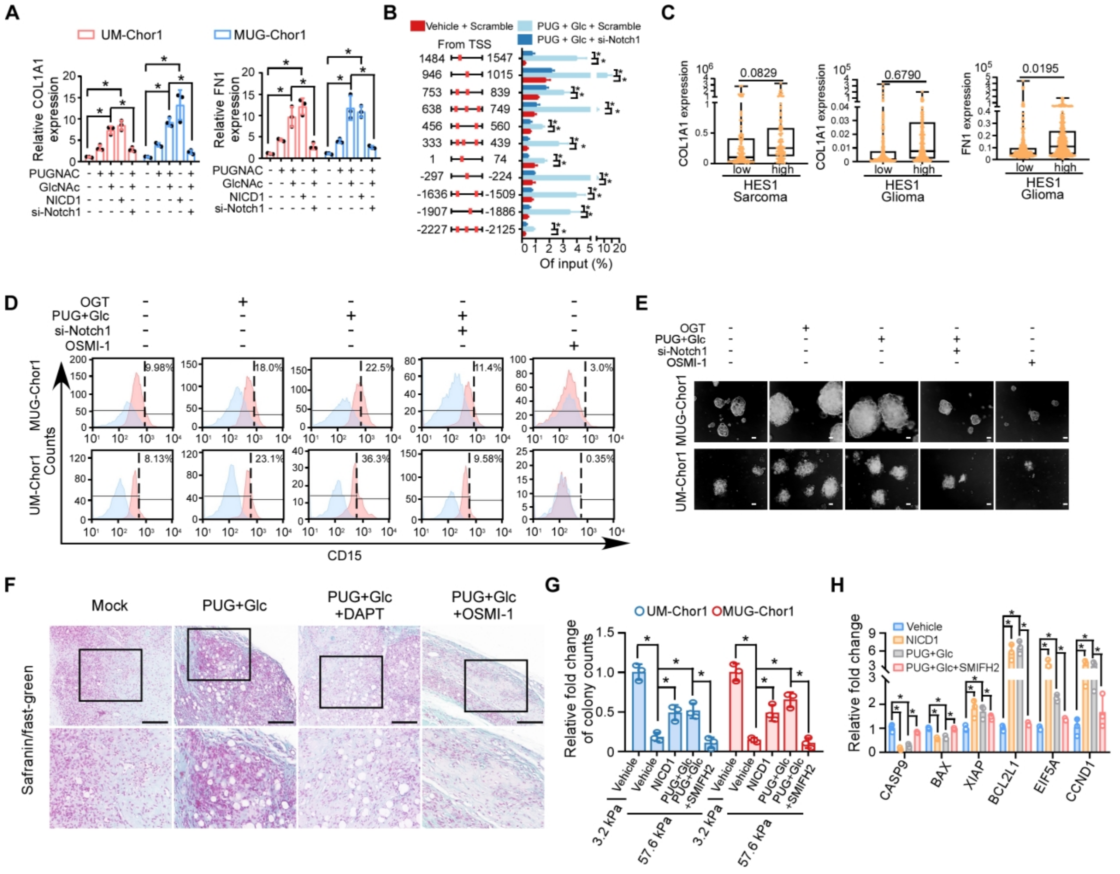

Supplemental Figure 3. NICD1 O-GlcNAcylation promotes tumor mechanical properties remodeling and tumor stemness. (A) qRT-PCR to assess the expression of COL1A1 and FN1 in response to NICD1 overexpression or PUGNAC and GlcNAc with or without NICD1 silencing. **(B)** ChIP to determine the interaction between RBP-Jκ and the promoter of *COL1A1*. The binding sites for RBP-Jκ was predicted by using ECR Browser. The site 0 indicates TSS. **(C)** Sarcoma or glioma patients were grouped according to HES1 expression, and the expression of COL1A1 and FN1, was analyzed. **(D and E)** Flow cytometry assay and sphere formation assay to determine CSC characteristics in chordoma cells in response to OGT overexpression, OSMI-1, PUGNAC and GlcNAc with or without NICD1 silencing. For E, scale bar: 50 µm. **(F)** Safranin O/Fast green staining to indicate extracellular proteoglycan in subcutaneous

tumors from Figure 3G. Scale bar: 200 μm. **(G** and **H)** Colony formation assay and qRT-PCR assessing whether SMIFH2 abrogate the effects of PUGNAC and GlcNAc on resistance to stiffness-induced death. Cells were cultured 7 d (G) or 48 h (H) respectively. Data in panel C is presented as min to max. Data in panels A, B, G, and H are presented as mean ± SD. Statistical analysis was performed using unpaired Student's *t* test (C) and one-way ANOVA (A, B, G, and H). *: $P < 0.05$.

Supplemental Figure 4

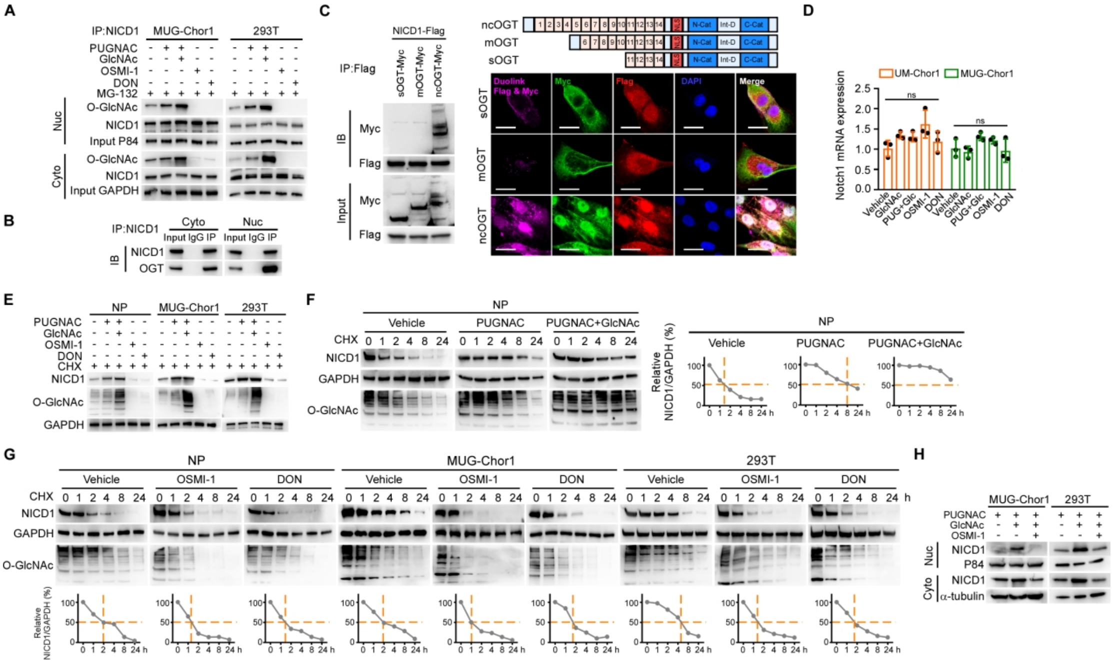

Supplemental Figure 4. O-GlcNAcylation at ANK and TAD domains of NICD1 leads to increased stability and transactivation activity. (A) Nuclear and cytoplasmic extracts were analyzed by IP to determine NICD1 O-GlcNAcylation. **(B)** The interaction between NICD1 and OGT in nucleus and cytoplasm respectively was determined by IP. **(C)** IP, Duolink PLA and IF to assess the interaction between NICD1 and three isoforms of OGT. Scale bar: 25 μm. **(D)** qRT-PCR to assess Notch1 mRNA level in chordoma cells in response to PUGNAC, GlcNAc, OSMI-1, and DON. **(E)** WB analysis in NP, MUG-Chor1, and 293T cells to determine the effects of O-GlcNAcylation on NICD1 protein level. **(F** and **G)** The effects of PUGNAC, GlcNAc, OSMI-1, and DON on the half-life of NICD1 were analyzed by WB. **(H)** NICD1 protein level in nucleus and cytoplasm in response to treatment of PUGNAC, GlcNAc, and OSMI-1 was identified respectively. Data in panel D is presented as

mean ± SD. Statistical analysis was performed using one-way ANOVA. ns: not significant.

Supplemental Figure 5

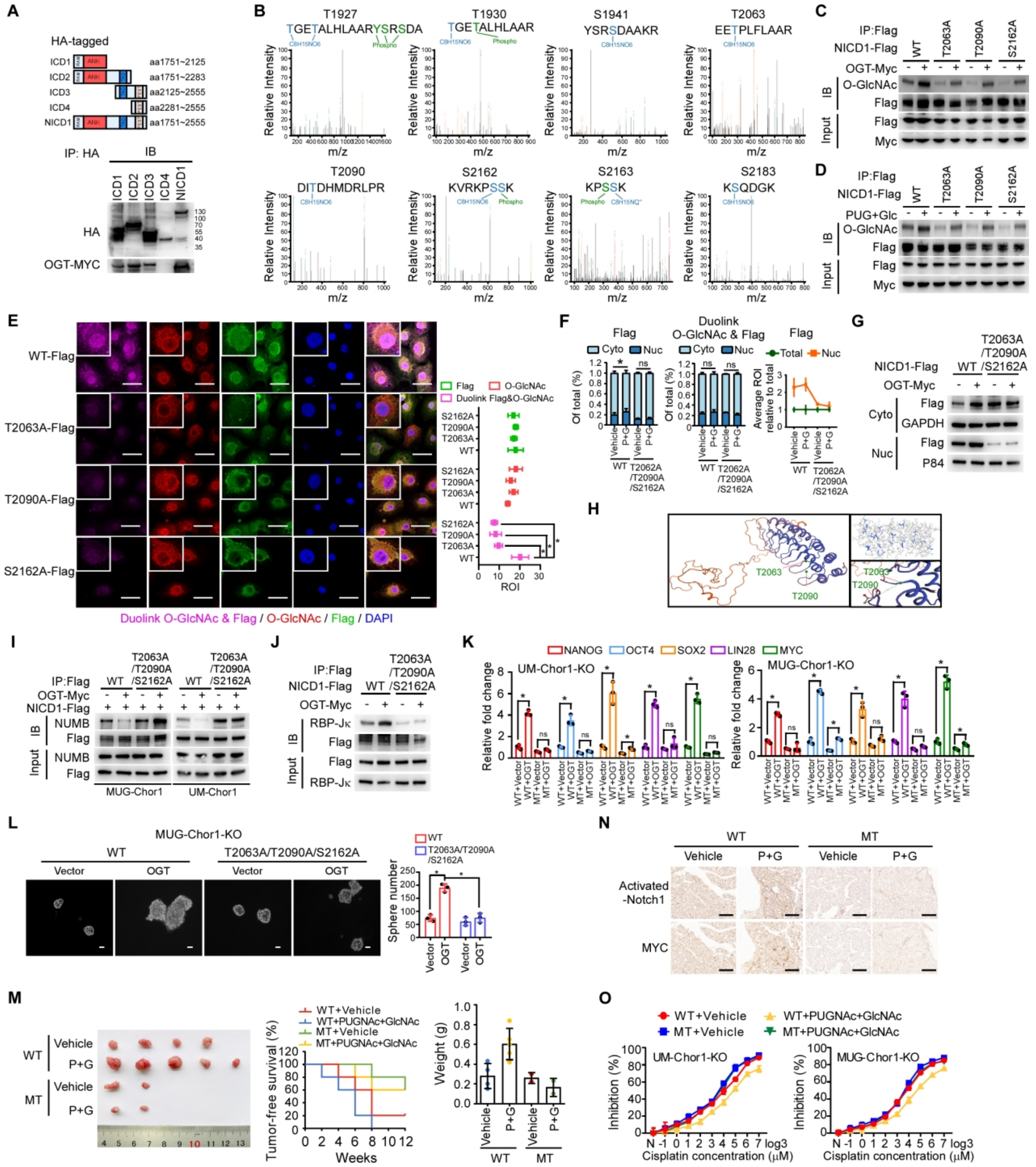

Supplemental Figure 5. T2063, T2090, and S2162 of NICD1 were identified as O-GlcNAcylation sites. (A) IP to assess the domains of Notch1 interacting with OGT-Myc. **(B)** The O-GlcNAcylation residues of NICD1 identified by IP and MS. **(C-E)** IP and Duolink PLA to determine O-GlcNAcylation of wild-type or mutant NICD1 in MUG-Chor1 cells with OGT overexpression or treatment of PUGNAC and GlcNAc. For E, scale bar: 50 μm. **(F)** Duolink PLA to assess NICD1

O-GlcNAcylation (Figure 4K). The ROI value of cytoplasmic and nuclear NICD1 O-GlcNAcylation was assessed respectively (middle panel). The average ROI of nuclear NICD1 O-GlcNAcylation relative to global NICD1 O-GlcNAcylation was analyzed. **(G)** Nuclear and cytoplasmic distribution of wild-type or mutated NICD1 in response to OGT overexpression was detected. **(H)** Structural modeling shows O-GlcNAc at T2063 and T2090 in the ANK domain. Protein modeling was performed using the online tool SwissModel (https://swissmodel.expasy.org/). **(I)** IP to determine the interaction between NUMB and wild-type or mutated NICD1. **(J)** IP to determine interaction between NICD1 and RBP-Jκ in stimulation with OGT overexpression. **(K)** Expression of CSC-related genes in cells with NICD1 knockout was assessed by qRT-PCR. **(L)** In chordoma cells with NICD1 knockout, sphere formation assay was performed when expressing wild-type or mutated NICD1 with or without OGT overexpression. **(M** and **N)** 10^7 MUG-Chor1-KO cells were subcutaneously inoculated into the nude mice (n = 5 per group). Tumors were treated with PUGNAC (10 mg/kg) and GlcNAc (10 mg/kg) every 3 d. Subcutaneous tumors, tumor-free survival time, and weight of the tumors were shown (M). Expression of activated-Notch1 and MYC in subcutaneous tumors were determined by IHC (N). Scale bar: 200 μm. **(O)** IC50 assay to assess the cytotoxicity of cisplatin on Notch1-KO cell lines when wild-type or mutant NICD1 was ectopically expressed with or without PUGNAc and GlcNAc. WT: wild-type NICD1. MT: T2063A/T2090A/S2162A NICD1. Data in panels E, F, K, L, M, and O are presented

as mean ± SD. Statistical analysis was performed using one-way ANOVA (E, K, and L) and Chi-square test (F). *: $P < 0.05$. ns: not significant.

Supplemental Figure 6

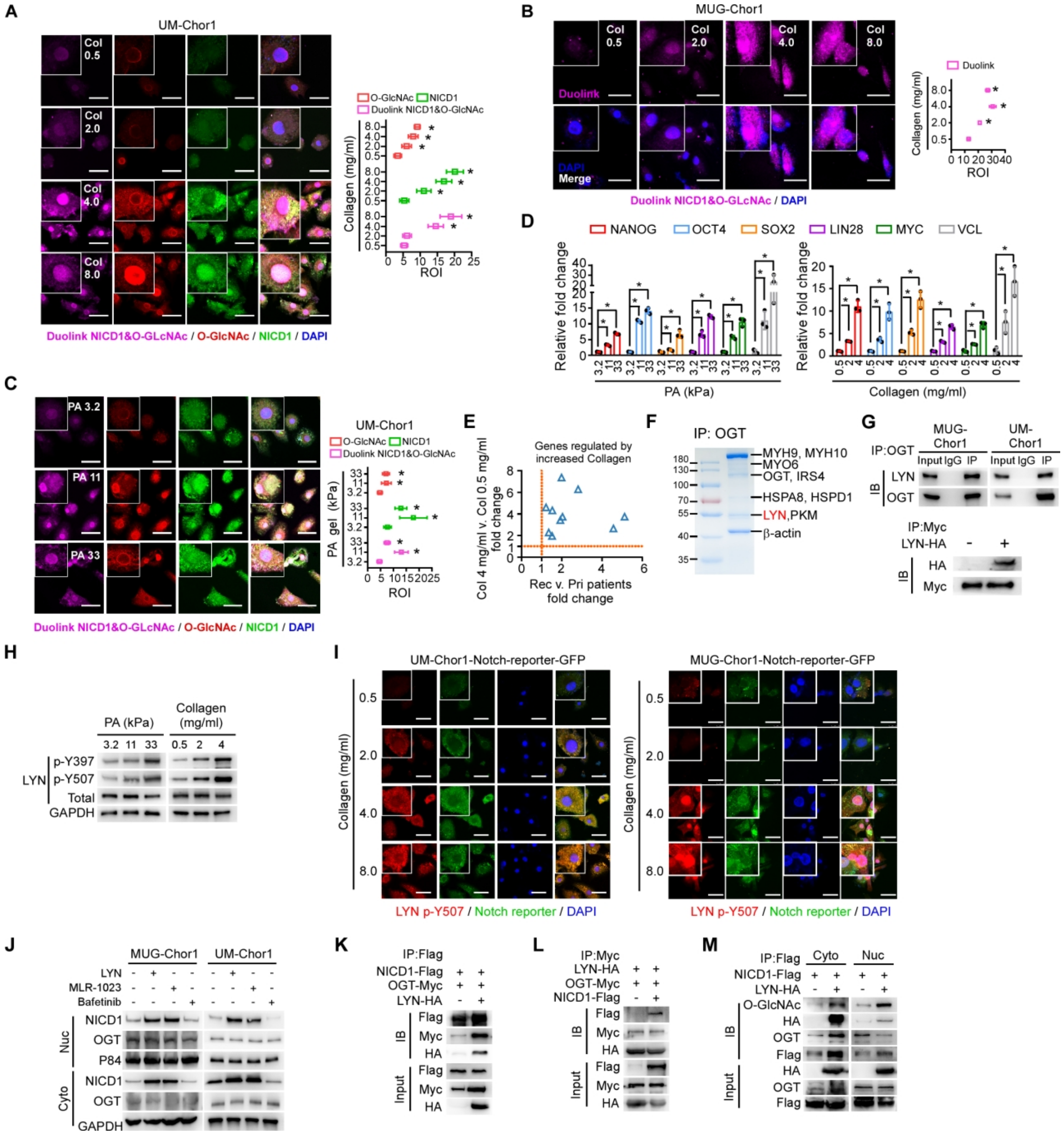

Supplemental Figure 6. Activation of LYN in response to stiff ECM mediates NICD1 O-GlcNAcylation. (A-C) Duolink PLA to determine NICD1 O-GlcNAcylation in chordoma cells cultured on substrates with increased stiffness. Scale bar: 50 μm. **(D)** Expression of CSC- and mechanical properties-related genes in chordoma cells in response to increased stiffness was detected by qRT-PCR. **(E)** Expression of genes regulated by increased stiffness was assessed by qRT-PCR in 10 paired primary and recurrent chordoma tissues, including HES1, HES5, HEY1, HEY2,

NRARP, MYC, NANOG, OCT4, VCL, and SOX2. The orange dotted line indicates the fold change of 1. **(F)** IP and MS were used to identify the proteins interacting with OGT. **(G)** To validate the OGT-LYN interaction, endogenous binding was assessed by Co-IP, and recombinant LYN-HA proteins were pulled down with OGT-Myc affinity gels followed by WB analysis. **(H)** WB to assess phosphorylation of LYN in response to increased stiffness in MUG-Chor1 cells. **(I)** IF to detect phosphorylation of LYN Y507 along with Notch signaling activity in response to increased stiffness. Scale bar: 50 μm. **(J)** WB to analyze nuclear and cytoplasmic NICD1 and OGT respectively in chordoma cells. **(K)** Interaction between NICD1 and OGT or LYN in response to LYN overexpression was identified by IP. **(L)** Interaction between OGT and LYN in response to NICD1 overexpression was identified by IP. **(M)** IP in cytoplasm and nucleus to detect NICD1 O-GlcNAcylation and interaction between NICD1 and LYN. Data in panels A, B, C, and D are presented as mean ± SD. Statistical analysis was performed using one-way ANOVA (A, B, C, and D). *: $P < 0.05$.

Supplemental Figure 7

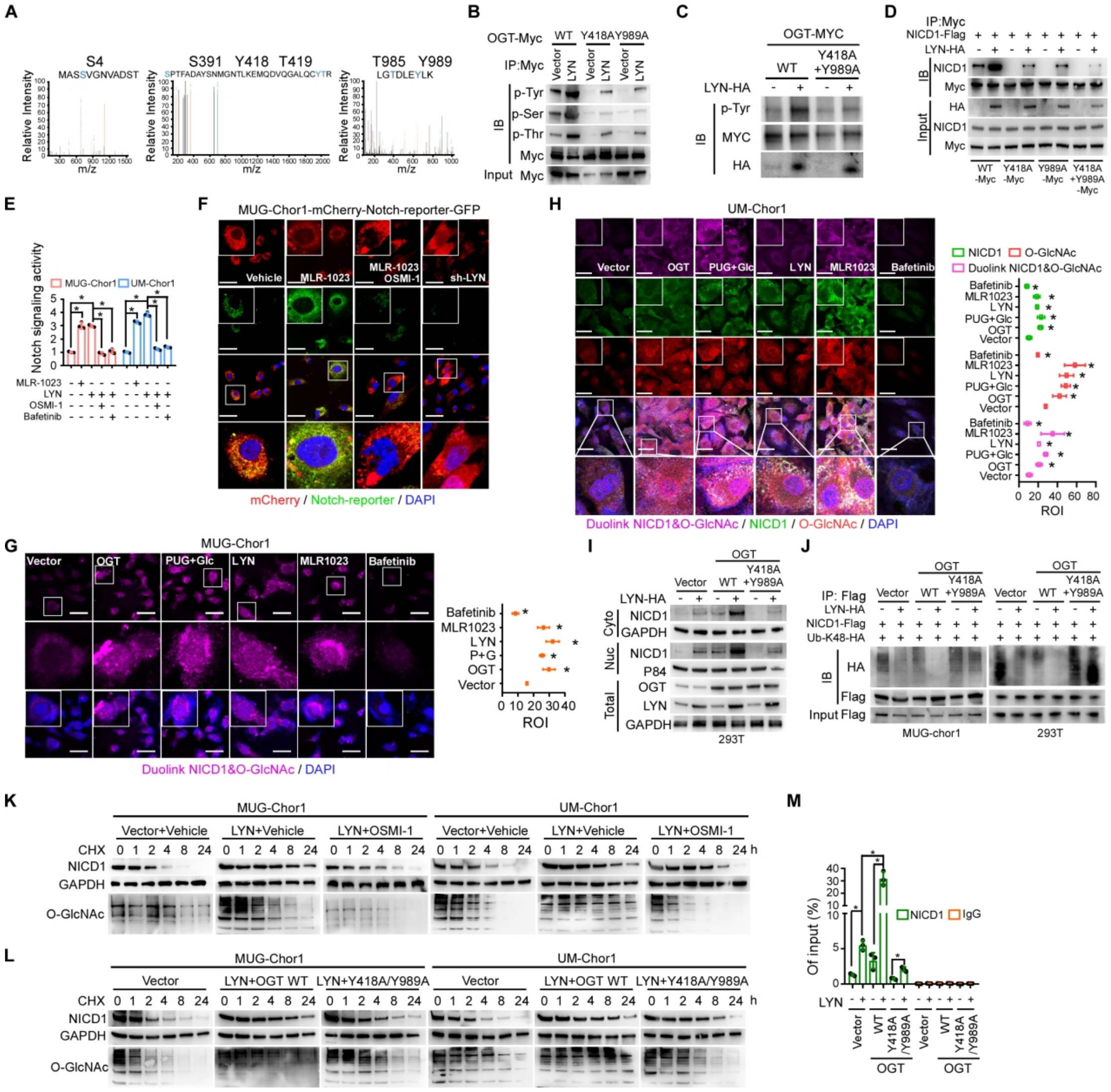

Supplemental Figure 7. LYN phosphorylates OGT and subsequently facilitates its catalytic activity. (A) Phosphorylated residues of OGT were identified by IP and MS. **(B)** IP to identify tyrosine, serine and threonine phosphorylation of wild-type (WT) and mutated OGT. **(C)** After purification of LYN-HA and wild-type or mutant OGT-MYC proteins, in vitro kinase assay was conducted to validate direct phosphorylation by LYN. **(D)** Interaction between NICD1 and wild-type or mutated OGT in stimulation to LYN overexpression was determined by IP. **(E and F)** The effects of LYN overexpression, MLR-1023, bafetinib, and OSMI-1 on Notch

signaling activity were determined by dual-luciferase reporter and IF. Scale bar: 50 μm. **(G** and **H)** Duolink PLA to show NICD1 O-GlcNAcylation, and IF to show NICD1 and global O-GlcNAcylation in chordoma cell lines in response to OGT and LYN overexpression, and treatment of PUGNAC, GlcNAc, MLR-1023, and bafetinib in chordoma cells. Scale bar: 50 μm. **(I)** Nuclear and cytoplasmic NICD1 protein was determined in cells expressing wild-type or mutated OGT. **(J)** IP to analyze the effect of wild-type or mutated OGT with or without LYN overexpression on ubiquitination of NICD1 in chordoma cells and 293T cells. **(K)** The effect of LYN overexpression with or without OSMI-1 treatment on the half-life of NICD1 was determined. **(L)** The effect of LYN overexpression with or without wild-type or mutated OGT ectopic expression on the half-life of NICD1 was determined. **(M)** ChIP analysis to identify the enrichment of NICD1 on *HES1* promoter in MUG-Chor1 cells expressing wild-type or mutated OGT with or without LYN overexpression. Data in panels E, G, H, and M are presented as mean ± SD. Statistical analysis was performed using one-way ANOVA (E, G, H, and M). *: $P < 0.05$.

Supplemental Figure 8

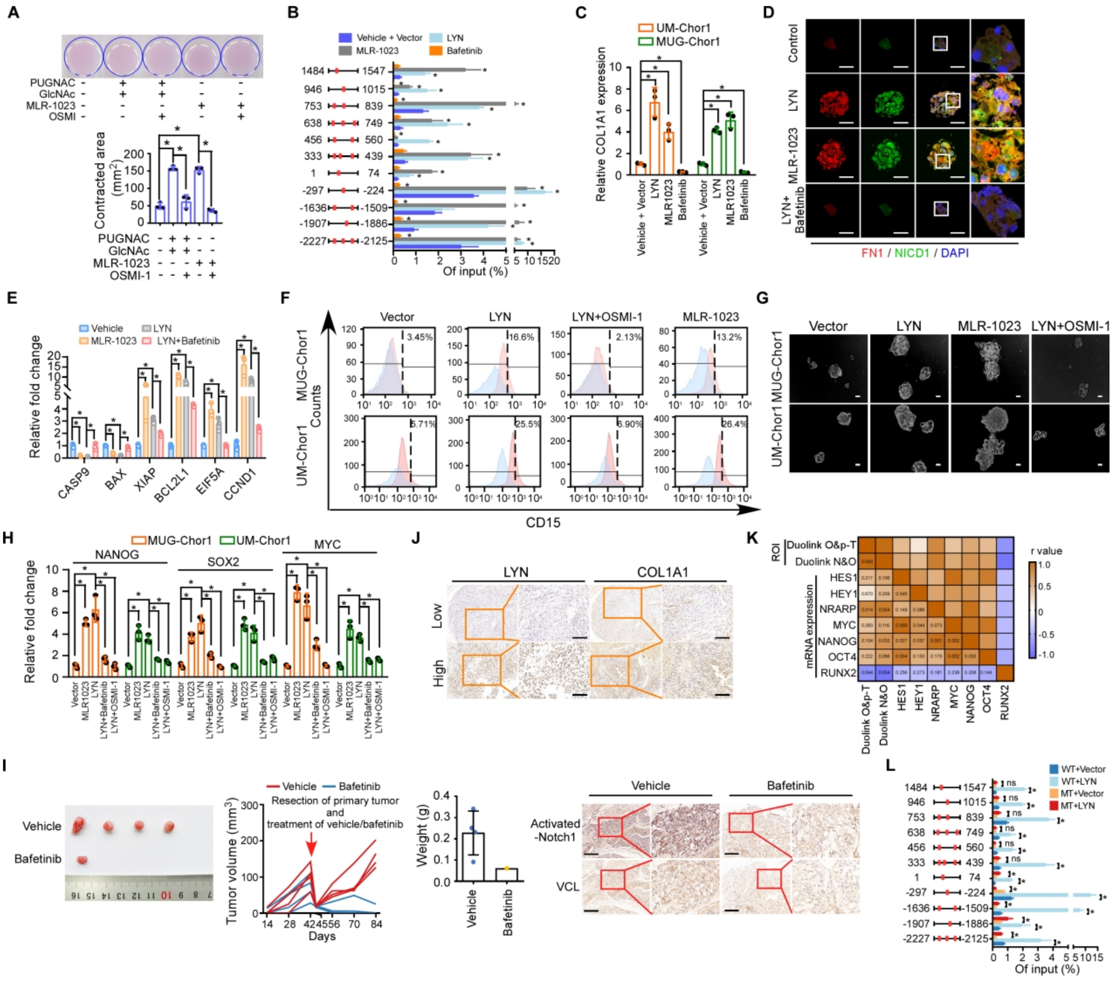

Supplemental Figure 8. LYN activity participates in regulation of mechanical properties and tumor stemness. (A) Collagen cell contraction assay to assess the effect of PUGNAC, GlcNAc, MLR-1023 (10 µM), and OSMI-1 (50 µM) on cellular contractility. **(B and C)** ChIP to analyze the enrichment of RBP-Jκ on *COL1A1* promoter and qRT-PCR to assess COL1A1 expression in response to overexpression of LYN, MLR-1023 (10 µM), and bafetinib (10 nM). The site 0 indicates TSS. **(D)** FN1 and NICD1 expression in spheroids developed from cells with overexpression of LYN, and treatment of MLR-1023 or bafetinib was assessed by IF. Scale bar: 50 µm. **(E)** Analysis of cell death by qRT-PCR under the indicated treatment conditions. **(F**

and **G)** Flow cytometry and sphere formation assays were performed in cells with treatment of MLR-1023 or OSMI-1, or LYN overexpression. Scale bar: 50 μm. **(H)** Expression of CSC-related genes was detected by qRT-PCR. **(I)** 10^7 MUG-Chor1 cells were subcutaneously inoculated into the nude mice (n = 5 per group). Tumors were treated with bafetinib (10 mg/kg) every 3 d. Subcutaneous tumors, tumor-free survival time, and weight of the tumors were shown. Expression of activated-Notch1 and VCL in subcutaneous tumors were determined by IHC. Scale bar: 200 μm. **(J)** IHC analysis to detect the expression of LYN and COL1A1 in chordoma grouped by LYN level. Scale bar: 100 μm. **(K)** The correlation between OGT tyrosine phosphorylation, NICD1 O-GlcNAcylation, and Notch signaling- or CSC-related genes. *P* values were presented in the box. **(L)** The enrichment of RBP-Jκ on *COL1A1* promoter was determined by ChIP. WT: wild-type NICD1 MT: T2063A/T2090A/S2162A NICD1. Data in panels A, B, C, E, H, I, and L are presented as mean ± SD. Statistical analysis was performed using one-way ANOVA (A, B, C, E, H, and L). *: $P < 0.05$. ns: not significant.

Supplemental Figure 9

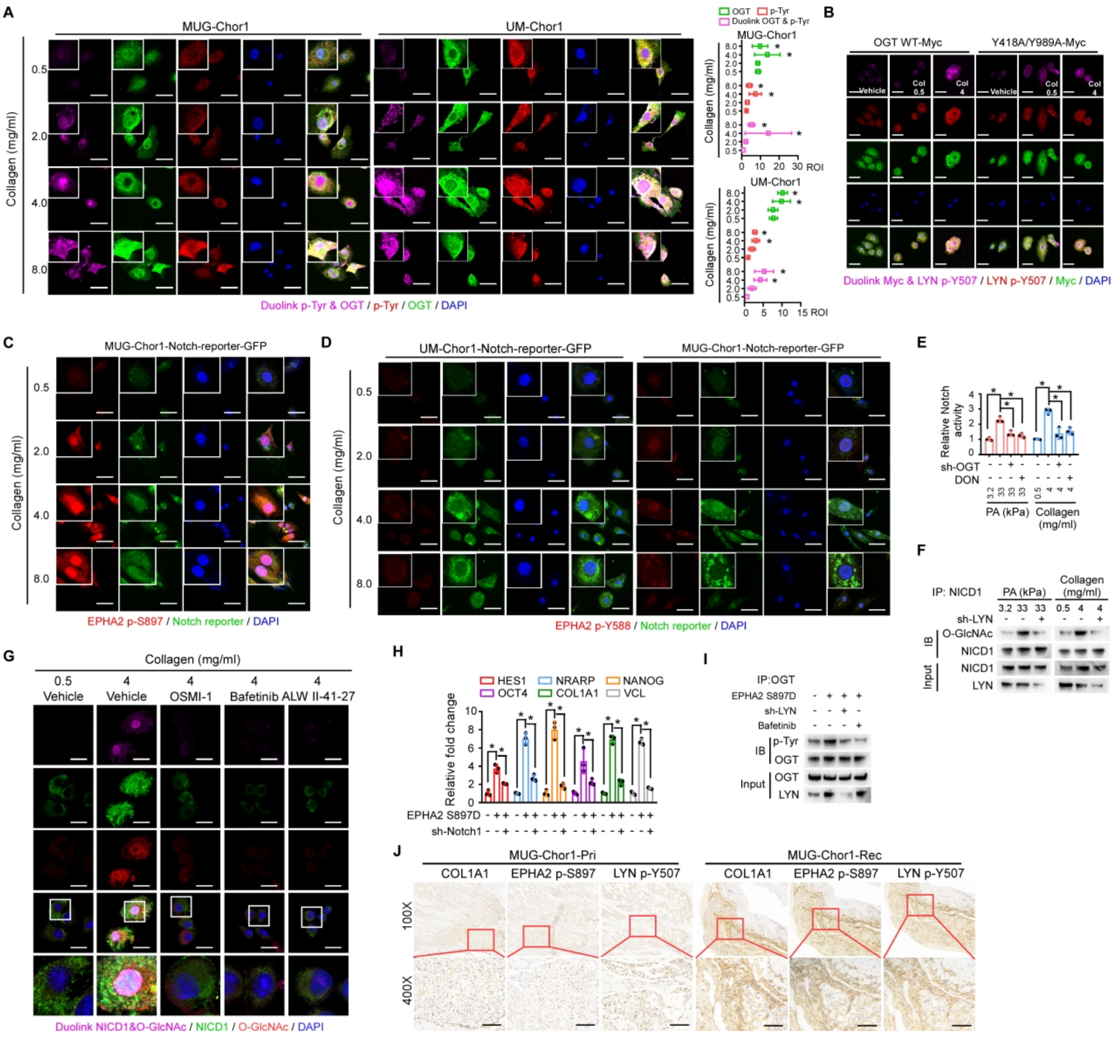

Supplemental Figure 9. EPHA2 perceives ECM stiffness to induce LYN phosphorylation. (A) Duolink PLA to detect OGT tyrosine phosphorylation in response to increased stiffness. Scale bar: 50 µm. **(B)** Interaction between wild-type (WT) or mutated OGT and Y507-phosphorylated LYN in response to increased stiffness was determined. Scale bar: 50 µm. **(C and D)** IF to assess the phosphorylation of EPHA2 S897 and Y588 as well as Notch activity in response to increased stiffness. Scale bar: 50 µm. **(E)** The effects of silencing OGT or DON on Notch signaling activity in MUG-Chor1 cells cultured on substrates with different stiffness were determined by dual-luciferase reporter. **(F)** The effects of silencing LYN on NICD1 O-GlcNAcylation in MUG-Chor1cells cultured on substrates with

different stiffness were determined by IP. **(G)** Duolink PLA to show NICD1 O-GlcNAcylation, and IF to show NICD1 and global O-GlcNAcylation in chordoma cell lines cultured on substrates with different stiffness in response to ALW II-41-27, OSMI-1, or bafetinib. Scale bar: 50 μm. **(H)** Expression of Notch downstream genes, CSC- and mechanical-related genes were assessed using qRT-PCR. **(I)** The effects of silencing LYN or inhibiting LYN kinase activity on EPHA2 S897D-induced phosphorylation of OGT tyrosine were detected by IP. **(J)** IHC in subcutaneous tumors developed from MUG-Chor1-pri and MUG-Chor1-rec cells. Scale bar: 100 μm. Data in panels A, E, and H are presented as mean ± SD. Statistical analysis was performed using one-way ANOVA (A, E, and H). *: $P < 0.05$.

Supplemental Figure 10

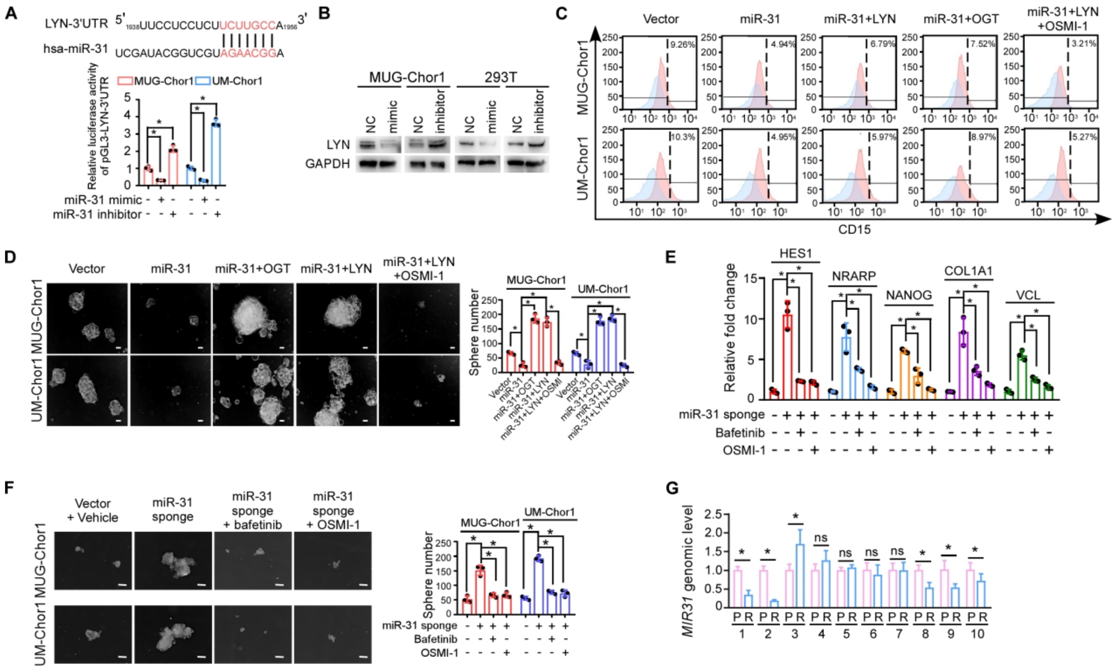

Supplemental Figure 10. Deletion of *MIR31* contributes to LYN upregulation in recurrent chordoma. (A) Luciferase assay of pGL3-LYN-3′-UTR reporter in the indicated cells, co-transfected with the indicated oligonucleotides. The sequence of miR-31 and LYN 3'UTR are shown. **(B)** LYN protein level was assessed in cells in stimulation of miR-31 mimic or inhibitor. **(C)** Flow cytometry to determine CSC-like properties. **(D)** Sphere formation was used to determine CSC-like properties. Scale bar: 50 μm. **(E)** Expression of Notch signaling-, CSC-, and mechanical properties-related genes was detected by qRT-PCR. **(F)** Sphere formation was used to determine CSC-like properties. Scale bar: 100 μm. **(G)** Level of genomic *MIR31* was assessed in 10 paired primary and recurrent chordoma tissues by utilizing qRT-PCR. Data in panels A, D, E, F, and G are presented as mean±SD. Statistical analysis was performed using unpaired Student's *t* test (G) and one-way ANOVA (A, D, E, and F). *: $P < 0.05$.

Supplemental Tables

Supplemental Table 1. Characteristics of patients with chordoma

	Total (N = 87)	
**Variables**	**No.**	**Percentage**
**Age (years)**		
Mean	45.59	
Median (range)	47 (1-79)	
**Gender**		
Female	26	29.89%
Male	61	70.11%
**Location**		
Sacrum	47	54.02%
Skull base	30	34.48%
Mobile spine	9	10.34%
Extra-axis	1	1.15%
**Pathological subtype**		
Conventional	79	90.80%
Chondroid	4	4.60%
Dedifferentiated	4	4.60%
**Clinical staging**		
I	55	63.22%
II	21	24.14%
III	2	2.30%
IV	9	10.34%
**T**		
T1	53	60.92%
T2	30	34.48%
T3	4	4.60%
T4	0	0.00%
**N**		
N0	0	0.00%
N1	1	1.15%
NX	86	98.85%
**M**		
M0	79	90.80%
M1	8	9.20%
**Pathological Grade (G)**		
G1	18	20.69%
G2	42	48.28%
G3	27	31.03%
G4	0	0.00%
**Adjuvant therapy**		
Embolism	1	1.15%

Ablation	8	9.20%
Targeted therapy	1	1.15%
No	77	88.51%
**Chemotherapy**		
Yes	2	2.30%
No	85	97.70%
**Radiotherapy**		
Yes	14	16.09%
No	73	83.91%
**Tumor size (major axis, cm)**		
Mean	5.95	
Median (range)	5.5 (1-13)	
**Recurrence**		
Yes	47	54.02%
No	30	34.48%
**Overall survival**		
Death	13	14.94%
Alive	74	85.06%
**Follow-up time (months)**		
Mean	59.98	
Median (range)	44 (6-305)	

Supplemental Table 2. Characteristics of matched primary and recurrent chordoma patient

No.	Loaction	Pathological subtype		Recurrence time (month)	Experimental type	Anti-tumor treatments
		Primary	Recurrent			
1	Skull base	Conventional	Conventional	7	IHC	
2	Sacrum	Conventional	Conventional	24	qPCR & IHC	
3	Sacrum	Conventional	Dedifferentiated	16	mRNA microarray	
4	Sacrum	Conventional	Conventional	11	mRNA microarray	Postoperation radiotherapy
5	Skull base	Conventional	Conventional	26	IHC	Postoperation radiotherapy
6	Sacrum	Conventional	Conventional	12	qPCR & IHC	
7	Sacrum	Conventional	Conventional	42	qPCR & IHC	
8	Sacrum	Dedifferentiated	Dedifferentiated	19	qPCR & IHC	
9	Skull base	Conventional	Conventional	8	IHC	
10	Mobile spine	Conventional	Conventional	18	IHC	Preoperative embolization & Postoperation radiotherapy
11	Sacrum	Conventional	Conventional	19	qPCR & IHC	
12	Skull base	Dedifferentiated	Dedifferentiated	54	IHC	
13	Skull base	Conventional	Conventional	61	IHC	Postoperation radiotherapy
14	Mobile spine	Conventional	Conventional	69	mRNA microarray & IHC	
15	Sacrum	Conventional	Conventional	27	qPCR & IHC	
16	Mobile spine	Conventional	Dedifferentiated	28	mRNA microarray	
17	Sacrum	Conventional	Conventional	18	mRNA microarray & IHC	
18	Sacrum	Conventional	Conventional	36	qPCR & IHC	
19	Sacrum	Conventional	Dedifferentiated	19	qPCR & IHC	
20	Sacrum	Conventional	Dedifferentiated	29	qPCR & IHC	
21	Sacrum	Conventional	Conventional	46	IHC	
22	Sacrum	Conventional	Conventional	76	qPCR & IHC	
23	Mobile spine	Conventional	Conventional	60	mRNA microarray	
24	Skull base	Conventional	Conventional	4	IHC	

Supplemental Table 3. Correlation between the sex, age, and protein level of NICD1

		NICD1 expression		*P*-value
		**Low**	**High**	
Sex	male	28	33	0.64
	female	14	12	
Age	< 50	28	23	0.19
	≥ 50	14	22	

Supplemental Table 4. IC50 Quantification of Supplemental Figure 1M

		IC50 (μM)
UM-Chor1	Vector	49.21475563
	NICD1	192.0179225
MUG-Chor1	Vector	57.68506337
	NICD1	173.0018852

Supplemental Table 5. Correlation between the sex, age, and protein level of NICD1 O-GlcNAc

		NICD1 O-GlcNAc expression		***P*-value**
		**Low**	**High**	
Sex	male	32	29	1.00
	female	13	13	
Age	< 50	28	23	0.52
	≥ 50	17	19	

Supplemental Table 6. IC50 Quantification of Supplemental Figure 5O

		IC50 (μM)
UM-Chor1-KO	WT+Vehicle	62.98808118
	WT+PUGNAc+GlcNAc	183.7725867
	MT+Vehicle	39.57798511
	MT+PUGNAc+GlcNAc	48.73873693
MUG-Chor1-KO	WT+Vehicle	63.24079451
	WT+PUGNAc+GlcNAc	173.6753848
	MT+Vehicle	46.78879752
	MT+PUGNAc+GlcNAc	60.78116713

Supplemental Table 7. Sense and antisense primers used for qRT-PCR

Name	Sequence (5'-3')
NOTCH1-Sense	GAGGCGTGGCAGACTATGC
NOTCH1-Antisense	CTTGTACTCCGTCAGCGTGA
HES1-Sense	TCAACACGACACCGGATAAAC
HES1-Antisense	GCCGCGAGCTATCTTTCTTCA
HES5-Sense	TGCTCAGCCCCAAAGAGAAA
HES5-Antisense	GAAGGCTTTGCTGTGCTTCA
HEY1-Sense	GTTCGGCTCTAGGTTCCATGT
HEY1-Antisense	CGTCGGCGCTTCTCAATTATTC
HEY2-Sense	AAGGCGTCGGGATCGGATAA
HEY2-Antisense	AGAGCGTGTGCGTCAAAGTAG
NRARP-Sense	TCAACGTGAACTCGTTCGGG
NRARP-Antisense	ACTTCGCCTTGGTGATGAGAT
CD133-Sense	CCATTGGCATTCTCTTTGAA
CD133-Antisense	TTTGGATTCATATGCCTTCTGT
SOX2-Sense	AACCCCAAGATGCACAACTC
SOX2-Antisense	GCTTAGCCTCGTCGATGAAC
NANOG-Sense	GATTTGTGGGCCTGAAGAAA
NANOG-Antisense	ATGGAGGAGGGAAGAGGAGA
MYC-Sense	CACCGAGTCGTAGTCGAGGT
MYC-Antisense	TTTCGGGTAGTGGAAAACCA
OCT4-Sense	GTGGAGGAAGCTGACAACAA
OCT4-Antisense	GGTTCTCGATACTGGTTCGC
LIN28-Sense	TGCGGGCATCTGTAAGTGG
LIN28-Antisense	GGAACCCTTCCATGTGCAG
AXIN2-Sense	CAACACCAGGCGGAACGAA
AXIN2-Antisense	GCCCAATAAGGAGTGTAAGGACT
LEF1-Sense	TGGATCTCTTTCTCCACCCA
LEF1-Antisense	CACTGTAAGTGATGAGGGGG

CCND1-Sense	TCCTCTCCAAAATGCCAGAG
CCND1-Antisense	GGCGGATTGGAAATGAACTT
SURVIVIN-Sense	CTTTCTCCGCAGTTTCCTCA
SURVIVIN-Antisense	TTGGTGAATTTTTGAAACTGGA
COL1A1-Sense	GAGGGCCAAGACGAAGACATC
COL1A1-Antisense	CAGATCACGTCATCGCACAAC
FN1-Sense	CGGTGGCTGTCAGTCAAAG
FN1-Antisense	AAACCTCGGCTTCCTCCATAA
VCL-Sense	CTCGTCCGGGTTGGAAAAGAG
VCL-Antisense	AGTAAGGGTCTGACTGAAGCAT
CASP9-Sense	CTTCGTTTCTGCGAACTAACAGG
CASP9-Antisense	GCACCACTGGGGTAAGGTTT
BAX-Sense	CCCGAGAGGTCTTTTTCCGAG
BAX-Antisense	CCAGCCCATGATGGTTCTGAT
XIAP-Sense	ACCGTGCGGTGCTTTAGTT
XIAP-Antisense	TGCGTGGCACTATTTTCAAGATA
BCL2L1-Sense	GAGCTGGTGGTTGACTTTCTC
BCL2L1-Antisense	TCCATCTCCGATTCAGTCCCT
EIF5A-Sense	GGACTTCGAGACAGGAGATGC
EIF5A-Antisense	TCATTCCTTTTGATGTTGGGGAC
GAPDH-Sense	GGAGCGAGATCCCTCCAAAAT
GAPDH-Antisense	GGCTGTTGTCATACTTCTCATGG
β-actin-Sense	CATGTACGTTGCTATCCAGGC
β-actin-Antisense	CTCCTTAATGTCACGCACGAT
MIR31-Sense	GTATTCTCCTGTAACTTGGAA
MIR31-Antisense	CTGGCATGCAGGTGGCCAT
Line1-Sense	AAAGCCGCTCAACTACATGG
Line1-Antisense	TGCTTTGAATGCGTCCCAGAG

Supplemental Methods

Click-iT O-GlcNAc enzymatic labeling and stoichiometric analysis of O-GlcNAcylated NICD1

Cell lysates were labeled by utilizing the Click-iT O-GlcNAc Enzymatic Labeling System (C33368, Invitrogen, Carlsbad, CA, USA). Modified proteins were detected by utilizing the Click-iT Biotin Protein Analysis Detection Kit protocol (Invitrogen). Biotinylated proteins were resolubilized in binding buffer (0.1 M phosphate, 0.15 M NaCl, 0.1% SDS, and 1% Nonidet P-40, pH 7.2). An appropriate amount of streptavidin resin (Thermo Fisher Scientific, Rockford, IL, USA) was added to incubate with the mixture overnight at 4°C. The streptavidin-bound complex was washed with binding buffer. Following the removal of supernatants, pellets were eluted by boiling with loading buffer (2% SDS, 10% glycerol, 2.5% 2-mercaptoethanol, and 62.5 mM Tris-HCl, pH 6.8). For quantifying the O-GlcNAc stoichiometry of individual proteins, the intensity of the total NICD1 protein band (Input) and the O-GlcNAcylated NICD1 protein band (Elution) were measured.

Reagents

PUGNAC (Abcam, Cambridge, UK) and UDP-GlcNAc (Sigma-Aldrich, St. Louis, MO, USA) was used to promote O-GlcNAc modification. OSMI-1 (Sigma-Aldrich) and 6-diazo-5-oxo-L-norleucine (DON) (Sigma-Aldrich) were utilized to inhibit O-GlcNAcylation. MLR-1023 (Selleck, Houston, TX, USA) was used to activate LYN kinase. Bafetinib (Selleck) was utilized to inhibit LYN kinase activity. SMIFH2 (Selleck) was used to inhibit actin polymerization.

Collagen gel preparation

Type I collagen gels were prepared using a modified protocol from Artym and Matsumoto (1). Briefly, to culture chordoma cells in collagen, desired number of cells were resuspended in 1×10^6 cells /ml in a mix of rat tail type I collagen (Corning, Corning, NY, USA), 0.02 N acetic acid, 10× DMEM, and 10× reconstitution buffer (0.2 M Hepes (GIBCO, San Jose, CA, USA) and 0.262 M $NaHCO_3$). Different density gels contained 0.5 mg/ml, 2 mg/ml, 4 mg/ml, and 8 mg/ml type I collagen respectively. 200 µl collagen solution was plated per well and allowed to polymerize at 37°C for 1 h. A second layer of 250 µl gel-cells solution was seeded on top and allowed to polymerize for 2 h at 37°C.

PA gel preparation

40% acrylamide (AM) (Sigma-Aldrich), 1% methylene bisacrylamide (Bis) (Sigma-Aldrich), 10% ammonium persulfate (APS) (Sigma-Aldrich), and 1 M HEPES buffer (pH 8.0) were prepared separately. The specific proportions of each component are detailed in the table below. The resulting solution was transferred to parallel adhesive plates with a 0.75 mm gap, and the mixture was allowed to polymerize at room temperature for 2 h, forming the gel. Following polymerization, the polyacrylamide hydrogel was immersed in 10 mM HEPES buffer and incubated for over 72 h to ensure it reached its saturated water content. The polyacrylamide gel was then cut into circular slices to facilitate cultivation in a 6-well plate. A 0.5 mg/mL solution of sulfosuccinimidyl 6-((4-azido-2-nitrophenyl) amino) hexanoate (sulfo-SNAPAH) (Sigma-Aldrich) was applied to the surface of the gel. The gel was exposed to a 365 nm UV lamp for 10 minutes to induce crosslinking, then washed several times with pH 8.0, 50 mM HEPES buffer to remove any residual crosslinking agent. Next, type I collagen (0.2 mg/mL) (Sigma-Aldrich) was added to the surface of

the modified polyacrylamide gel. The gel was incubated overnight at 4°C to allow the collagen to crosslink onto the gel surface. After incubation, uncrosslinked collagen was removed by washing the gel with sterile PBS. Prior to cell inoculation, the cells were incubated in medium at 37°C for 1 h. The detailed formula is as follows:

Stiffness	40 % AM/ml	1 % Bis/ml	H_2O/ml	10 % APS/μl	TEMED/μl
3.2 kPa	1.25	0.15	3.55	50	5
11.0 kPa	1.25	0.5	3.25	50	5
33.0 kPa	1.25	1.5	2.25	50	5
57.6 kPa	1.25	2.5	1.25	50	5

In vitro UDP-Glo ™ Glycosyltransferase assay to assess OGT activity

The Promega UDP-Glo™ Glycosyltransferase Assay was used to assess the OGT activity in vitro (2). According to the manufacturer's instructions, wild-type and mutated OGT recombinant proteins were purified from chordoma cells with indicated treatment. Luminescence was recorded 1 h after adding the UDP Detection Reagent which correlates to the amount of UDP produced by the OGT. The OGT activity was evaluated through monitoring UDP formation in glycosyltransferase reactions by luminescence. Specifically, OGT reactions were carried out in 100 μL OGT reaction buffer containing 0.5 mM UDP-GlcNAc, 6 μg purified / 60 μg lysate enzyme, and 100 μM peptide. Reactions were incubated at room temperature for 1 h. Luminescence was recorded using a microplate reader. Values represent the mean of three replicates.

Immunoprecipitation and protein purification

Lysates were prepared from 3×10^7 293T cells transfected with Flag-, HA- or Myc-tagged full-length or truncated NICD1, LYN or OGT in an NP-40-containing

lysis buffer supplemented with protease inhibitor cocktail (Beyotime Biotechnology, China), and then immunoprecipitated with Flag, HA or Myc affinity agarose (Sigma-Aldrich) overnight at 4°C. Beads containing affinity-bound proteins were washed six times with IP wash buffer (150 mM NaCl, 10 mM HEPES, pH 7.4, 0.1% NP-40). The eluted proteins were denatured and separated on SDS-polyacrylamide gels analyzed by WB and stained with Coomassie blue; the indicated bands were subjected to mass spectrometry (MS) analysis.The purification of recombinant proteins NICD1-Flag, LYN-HA or OGT-Myc was performed via IP as described previously (3).

MS analysis of NICD1 O-GlcNAcylation sites and OGT phosphorylation sites

Lysates of Chordoma cells expressing NICD1-Flag or OGT-Myc were immunoprecipitated by FLAG or Myc affinity agarose (Sigma-Aldrich) overnight at 4°C. Beads containing affinity-bound proteins were washed six times with IP wash buffer (250 mM NaCl, 10 mM HEPES, pH 7.4, 0.1% NP-40), followed by elution with 1 M glycine (pH 3.0) twice. Recombinant NICD1 or OGT proteins were analyzed by MS analysis. The eluted proteins were denatured, separated and analyzed by MS analysis (Applied Protein Technology, China). Modification of O-GlcNAc (Ser/Thr) and phosphorylation (Ser/Thr/Tyr) was identified (4).

mRNA Microarray Profiling of Paired Chordoma Specimens

Total RNA was extracted from formalin-fixed paraffin-embedded (FFPE) tumor sections of 6 matched primary-recurrent chordoma pairs using the RecoverAll™ Total Nucleic Acid Isolation Kit (Cat#AM1975, Ambion). RNA integrity was verified through spectrophotometry (NanoDrop ND-2000) and electrophoretic analysis

(Agilent Bioanalyzer 2100), with all samples meeting the quality threshold (RIN ≥ 6.0). Total RNA samples were subsequently processed by Shanghai Biotechnology Corporation for all downstream experiments.

Amplification and biotinylation were performed using the Ovation FFPE WTA System (Cat#3403, NuGEN) and FL-Ovation™ cDNA Biotin Module V2 (Cat#4200, NuGEN) according to manufacturer protocols. Fragmented biotinylated cRNA (5 μg per sample) was hybridized to Affymetrix GeneChip® Human Transcriptome Arrays for 18 hr at 45°C in a GeneChip® Hybridization Oven 645 (Cat#00-0331-220V, Affymetrix) using the GeneChip® Hybridization, Wash and Stain Kit (Cat#900720, Affymetrix).

Post-hybridization processing was conducted on a Fluidics Station 450 (Cat#00-0079, Affymetrix) following NuGEN's FL-Ovation™ protocol. Arrays were scanned with the GeneChip® Scanner 3000 (Cat#00-0212, Affymetrix), and raw data were acquired through Command Console Software 4.0 (Affymetrix).

Raw CEL files were normalized with the Robust Multi-array Average (RMA) using R package Affy (version 1.70.0). Quality control was performed by assessing signal distribution uniformity through boxplot visualization and verifying 3'/5' ratios of housekeeping genes (Beta-actin ≤ 15.5, GAPDH ≤ 9.85) to confirm RNA integrity in FFPE-derived samples. Differential expression analysis identified genes with $|\log_2$ fold-change$| \geq 1$ and Student’s t-test $P < 0.05$ (Benjamini-Hochberg FDR-adjusted), with results visualized via scatter plots of log2-transformed signals. Functional enrichment of differentially expressed genes was conducted using Sangerbox (http://sangerbox.com/tool.html) to evaluate GO terms and KEGG pathways, where significance was further prioritized by enrichment factor values calculated as the ratio of observed-to-expected gene frequencies in each term.

Chromatin immunoprecipitation assay and Chromatin immunoprecipitation sequencing

For ChIP analysis, SimpleChIP® Plus Enzymatic Chromatin IP Kit was utilized (Cell Signaling Technology, Danvers, MA, USA). 5×10^7 MUG-Chor1 cells were cultured and harvested for cross-linking and sheared by sonication. The resultant chromatin fraction was immunoprecipitated using 10 μg antibodies against cleaved-Notch1 (4147, Cell Signaling Technology), RBP-Jκ (ab317048, Abcam), Flag (F7425, Sigma-Aldrich), and negative control anti-IgG (AP112, Sigma-Aldrich). After reversing the cross-links with NaCl and removing proteins with proteinase K, enriched DNA fragments were purified and isolated via phenol/chloroform extraction and ethanol precipitation. The final DNA pellets were then subjected to Chromatin immunoprecipitation sequencing (Novogene, China) or real-time quantitative PCR with the indicated specific primers.

Gene set enrichment analysis (GSEA) and microarray data deposition

The Cancer Genome Atlas (TCGA) sarcoma and glioma datasets were downloaded to identify the association of NRARP, HES1 or Notch signaling activity with stemness-, Glycosylation- or ECM remodeling-related gene signatures using the GSEA software, respectively. In addition, total RNAs from chordoma tissues were collected for mRNA-sequencing analysis following standard protocol.

Primary sphere formation

Tumor spheres were cultured according to a previous report (5). Indicated cells (5×10^3), seeded in ultra-low adherent 6-well plates (Corning), were cultured in

DMEM/F12 serum-free medium (Invitrogen) supplemented with 2 % of B-27 (GIBCO, Grand Island, NY, USA), 20 ng/ml of EGF (BD Biosciences, San Jose, CA, USA), 20 ng/ml of bFGF (GIBCO), and 4 mg/mL insulin (Sigma-Aldrich) to form tumor spheres. Nutrient supplemented medium was added for the growth of spheres every 2 d for 10 d. Cell spheres were photographed and counted under 200× magnification.

To detect expression of COL1A1 and FN1, the spheroids were then collected and fixed with 4% paraformaldehyde. After static fixation, gently aspirate the supernatant. Subsequently, basic culture medium was added and allow for natural sedimentation. Pipette 50 μl of a completely dissolved agarose solution to resuspended the tumor spheroids, placed on ice for 30 minutes until it solidifies. The solidified agarose blocks were dehydrated in a gradient ethanol series and embedded in Paraffin. Sections were prepared from the paraffin-embedded blocks, followed by IF staining was performed, and images were taken with a Leica SP8 confocal microscope.

Flow cytometry

1×10^6 cells were used for flow cytometry analysis. The primary antibody used was anti-CD15-APC (dilution 1:20, 323008, BioLegend, San Diego, CA, USA). This experiment was performed according to the manufacturer's instructions.

Half maximal inhibitory concentration (IC50) assay

Tumor cells (1×10^4/well) were seeded in 0.2 mL of medium/well in 96-well plates and incubated for 24 h at 37°C in a 5% CO_2 incubator. The medium in the wells contained a series of dilutions of cisplatin (0, 3^{-1}, 3^0, 3^1, 3^2, 3^3, 3^4, 3^5, 3^6, and 3^7 M) (Sigma-Aldrich). After 48 h of incubation, 100 g MTT (3-(4, 5-dimethyl

thiazol-2yl)-2, 5-diphenyl tetrazolium bromide) (MedChemExpress, Monmouth Junction, NJ, USA) was added to each well and was further incubated at 37°C for 4 h. After incubation, the medium was removed and 180 µL DMSO (solubilizing reagent) (Sigma-Aldrich) was added to each well and mixed for 15 minutes. The suspension OD (optical density) values were read at 490 nm. Measurements were performed and the concentration required for IC50 was determined graphically. A standard graph was plotted by taking concentration of the drug in X axis and relative cell viability on the Y axis.

RNA extraction and qPCR

Total RNA from cultured cells and frozen surgical chordoma tissues was isolated with TRIzol reagent (Invitrogen) as instructed. cDNA was synthesized from 2 µg of total RNA with random primers with the use of the Gene Expression Assays (Promega, Madison, WI, USA) and analyzed with Biorad CFX Manager 3.1 software. Expression of mRNAs was assessed based on the threshold cycle (CT), and relative expression levels were calculated as $2^{-[(\text{Ct of mRNA}) - (\text{Ct of }\beta\text{-actin}]}$ after normalization to β-actin expression. Experiments were performed at least three times, with triplicate replicates. qRT-PCR primers of miR-31 were purchased from Ribo (Guangzhou, China). Sense and antisense primers used for qRT-PCR were listed in Supplementary table 7.

Primary antibodies

Primary antibodies used for Western blotting analysis were: anti-cleaved Notch1 (4147, Cell Signaling Technology), anti-Notch1 (3608, Cell Signaling Technology), anti-p-Tyr (9411, Cell Signaling Technology), anti-O-Linked N-Acetylglucosamine

(ab2739, Abcam), anti-OGT (ab177941, Abcam), anti-Flag (F7425, F3165, Sigma-Aldrich), anti-HA (H6908, H9658, Sigma-Aldrich), anti-Myc (2276, Cell Signaling Technology), anti-LYN (ab32398, Abcam), anti-p-Ser (SC-81514, Santa Cruz Biotechnology, Dallas, TX, USA), anti-p-Tyr (ab10321, Abcam), anti-LYN p-Y397 (ab226778, Abcam), anti-LYN p-Y507 (ab 33914, Abcam), anti-VCL (A2752, ABcolonal, China), anti-EPHA2 (6997, Cell Signaling Technology), anti-EPHA2 p-Y588 (AF7280, Affinity Biosciences, Cincinnati, OH, USA), anti-EPHA2 p-S897 (AF7279, Affinity Biosciences). Blotted membranes were stripped and re-blotted with anti-p84 (ab131268, Abcam), anti-GAPDH (2118S, Cell Signaling Technology), and anti-α-Tubulin (3873, Cell Signaling Technology) used as loading controls.

In vitro protein ubiquitination assay

Cells were transfected with various combinations of plasmids or siRNAs, or treated with indicated drugs, along with HA-tagged K48-linked ubiquitin (Ub-K48-HA). At 24 h after transfection, the cells were treated with MG132 (10 μM) for 6 h, and the whole-cell lysates were subjected to IP for exogenous Flag-tagged NICD1 proteins. The levels of NICD1 ubiquitination were detected by immunoblotting with anti-HA antibody (H6908, H9658, Sigma-Aldrich).

Plasmids, virus production and transfection

The open reading frames (ORFs) of NICD1 were generated by PCR amplification and subcloned into the pSin-EF2 lentiviral vectors (Addgene, Watertown, MA, USA) with different antibiotic resistance genes. Various deletion mutants of HA-tagged NICD1, NICD1 mutants, and OGT mutants were subcloned into retroviral transfer plasmid PQCXIP-puro (Clontech Laboratories, Mountain View, CA, USA) and

HA-tagged ubiquitin, were subcloned into a pcDNA 3.1 vector. OGT and LYN expression plasmid was purchased from Sino Biological (Beijing, China). HA-tagged K48-linked ubiquitin was subcloned into a pcDNA 3.1 vector. 3'-UTR of LYN was amplified into the downstream of the luciferase gene in a pGL3 control vector (Promega). Notch1-GFP-reproter construction was generated by cloning the DNA binding motif of NICD1 (CBF1/RBP-Jκ binding site: 4×CCGTGGGAAAAATTT) on the 7TFP (Addgene).

For depletion of Notch1, two human shRNA sequences were cloned in to pSuper-retro-puro vectors. All siRNA oligonucleotides, miRNA mimics, and miRNA inhibitors were purchased from Ribo (Guangzhou, China).

Stable cell lines were generated via retroviral or lentiviral infection and selected with appropriate antibiotics for 10-14 d. Transfection of plasmids or RNA oligonucleotides was performed using Lipofectamine 3000 reagent (Invitrogen) for luciferase reporter assays and molecular assays.

Dual-luciferase reporter assay

The Notch signaling activity was measured by dual-luciferase reporter assay (Promega) of Notch1 signaling reporter, which is constructed by cloning the DNA binding motif of NICD1 (CBF1/RBP-Jκ binding site: 4×CCGTGGGAAAAATTT) into pGL3-Basic plasmid as a promoter of Firefly Luciferase gene (Promega). Renilla Luciferase reporter (TK plasmid) was used as an internal control. Relative luciferase activity (Firefly Luciferase/Renilla Luciferase) of each treatment is calculated as the Notch signaling activity. The effects of miR-31 mimic and inhibitor on LYN was measured by cloning 3'-UTR of LYN into the downstream of the luciferase gene in pGL3 control vector (Promega). Renilla Luciferase reporter was also used as an

internal control. Cells were seeded in triplicate in 24 or 48-well plates and allowed to settle for 24 h. The indicated plasmids plus 10 ng pRL-TK renilla plasmid were transfected into the cells using the Lipofectamine 3000 reagent (Invitrogen). 48 h after transfection, dual-luciferase reporter assays were performed using a Dual Luciferase Reporter Assay Kit (Promega) according to the manufacturer's protocol.

Immunocytochemistry

IHC assays in chordoma tissues were performed and quantified according to our previous report (3). The degree of immunostaining of the indicated proteins was evaluated and scored by two independent observers as previously described, scoring both the proportions of positively stained tumor cells and their staining intensities. Scores representing the proportion of positively stained tumor cells was graded as: 0 (no positive tumor cells), 1 (< 10%), 2 (10-50%), and 3 (> 50%). The intensity of staining was determined as: 0 (no staining), 1 (weak staining = light yellow), 2 (moderate staining = yellow brown), and 3 (strong staining = brown). The staining index (SI) was calculated as the product of the staining intensity × the percentage of positive tumor cells, resulting in scores as 0, 1, 2, 3, 4, 6, and 9. Cutoff values for high and low expression levels of proteins of interest were chosen based on a measurement of heterogeneity using the log-rank test with respect to overall survival. The optimal cut-off for NICD1, OGT, MYC, HES1, O-GlcNAc, and LYN expression was identified according to the median value.

Immunofluorescence

Cells seeded on coverslips were fixed in 4% paraformaldehyde for 15 min at room temperature and then permeabilized with methyl alcohol for 10 min at room

temperature. Non-specific binding was blocked with 3% bovine serum albumin (Sigma-Aldrich) in PBS for 30 min before incubation with a primary antibody at 4°C overnight. Secondary antibodies (Rhodamine, 111-025-003; FITC, 111-095-003, 315-095-003; Alexa Fluor 647, 111-605-003; Jackson ImmunoResearch Laboratories, West Grove, PA, USA) were applied for 1 h at room temperature, followed by DAPI. After extensive washing between each step, coverslips were mounted onto microscopy slides with antifade Solution. Images were captured by using confocal (Leica SP8, Germany).

Nuclear and cytoplasmic extraction

A NE-PER Nuclear and Cytoplasmic Extraction Kit (Thermo Fisher Scientific) was used, and assays were performed according to the manufacturer's instructions.

Reference:

1. Artym VV, et al. Imaging cells in three-dimensional collagen matrix. *Curr Protoc Cell Biol*. 2010;Chapter 10:10-18.
2. Engel L, et al. Utility of Bioluminescent Homogeneous Nucleotide Detection Assays in Measuring Activities of Nucleotide-Sugar Dependent Glycosyltransferases and Studying Their Inhibitors. *Molecules*. 2021;26(20):6230.
3. Liu L, et al. An RFC4/Notch1 signaling feedback loop promotes NSCLC metastasis and stemness. *Nat Commun*. 2021;12(1):2693.
4. Wang Y, et al. O-GlcNAcylation destabilizes the active tetrameric PKM2 to promote the Warburg effect. *Proc Natl Acad Sci U S A*. 2017;114(52):13732-13737.
5. Liu L, et al. MTNR1B loss promotes chordoma recurrence by abrogating melatonin-mediated beta-catenin signaling repression. *J Pineal Res*. 2019;67(2):e12588.
