# Supplementary material for: Notch1 O-GlcNAcylation drives tumor stemness and mechanoadaptation to a stiff microenvironment and promotes chordoma recurrence
Source: J Clin Invest. 2026 Feb 3;136(6):e194378. doi: 10.1172/JCI194378 (PMC12987655; doi:10.1172/JCI194378)

Fig 2B

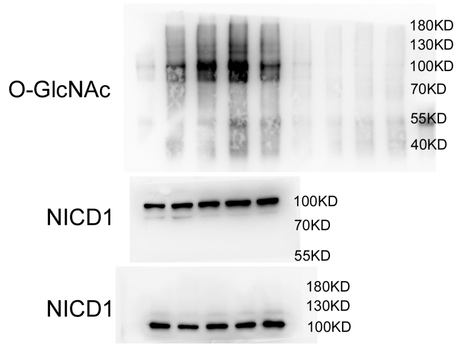

Fig 2C

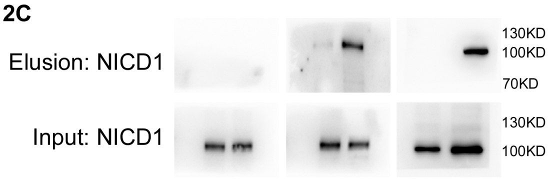

Fig 4A

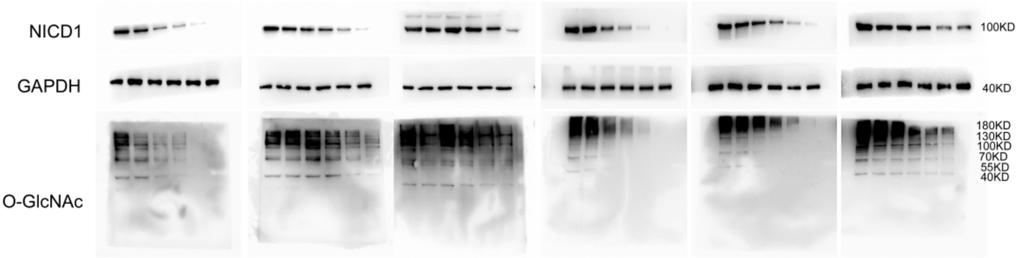

Fig 4B

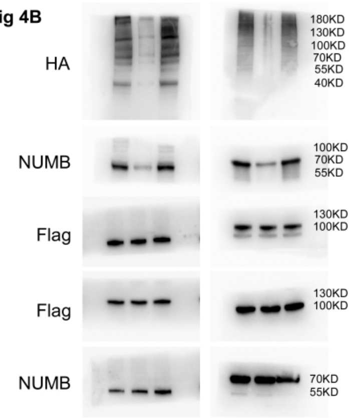

Fig 4C

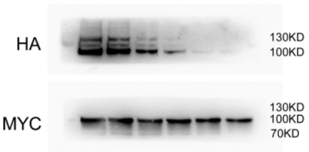

Fig 4I

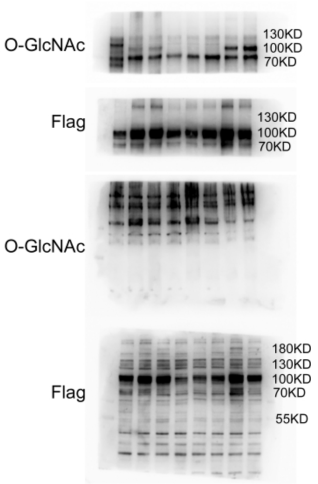

Fig 4J

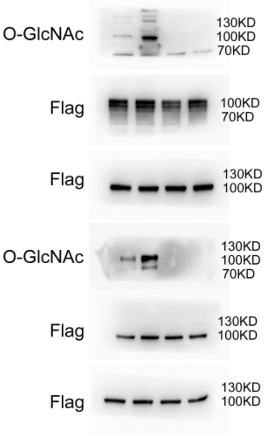

Fig 4L

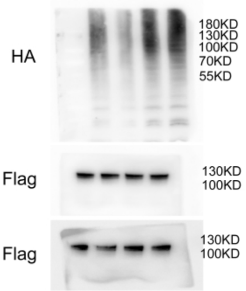

Fig 5B

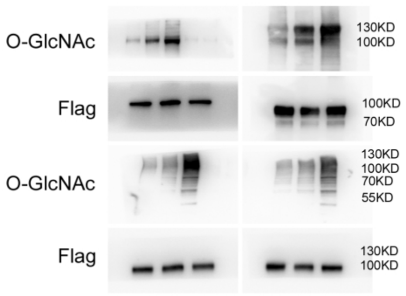

Fig 5D

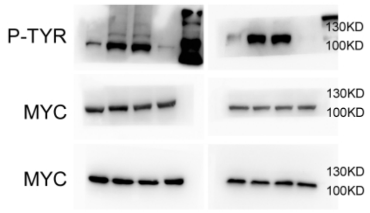

Fig 5E

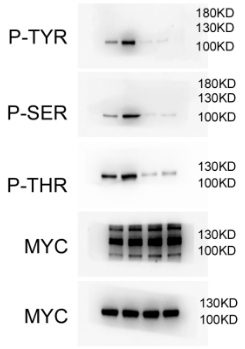

Fig 5H

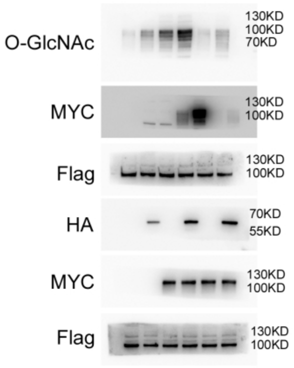

Fig 5I

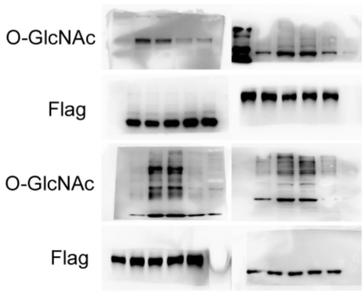

Fig 5J

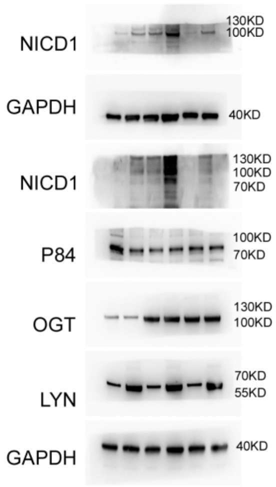

Fig 6A

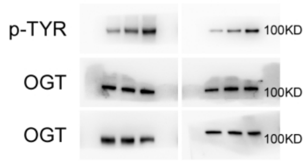

Fig 6C

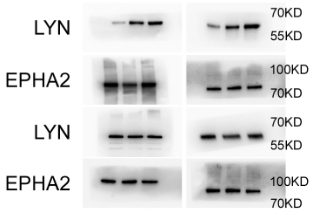

Fig 6D

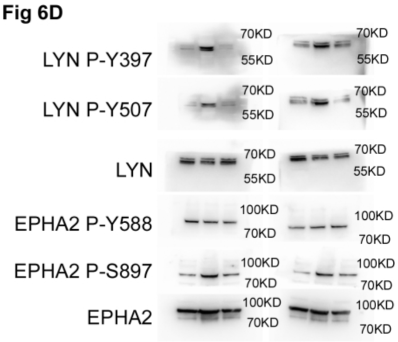

Fig 6F

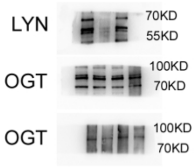

Fig 6G

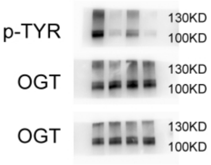

Fig 6H

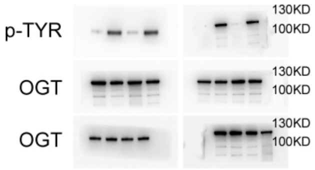

Fig 6J

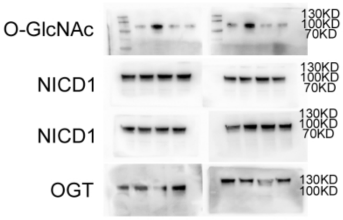

Fig 6K

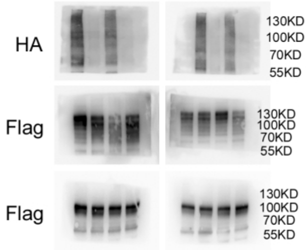

Fig 7D

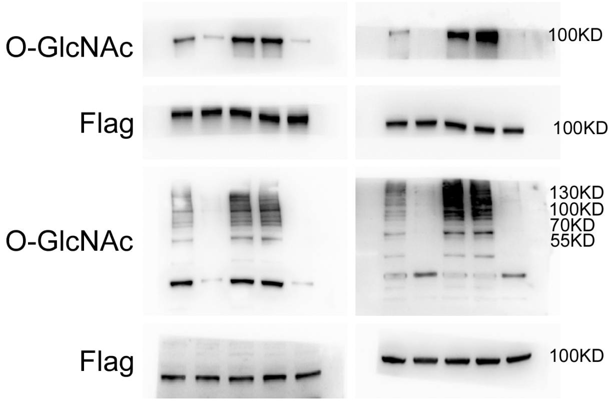

Fig S2A

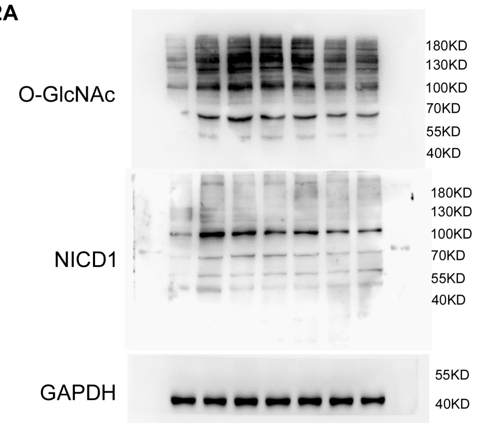

Fig S2C

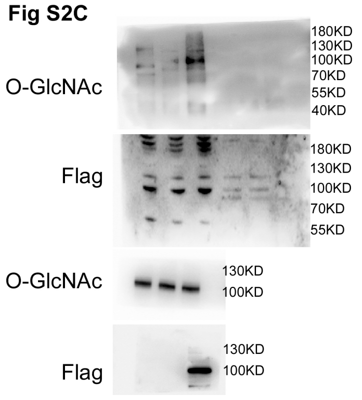

Fig S2D

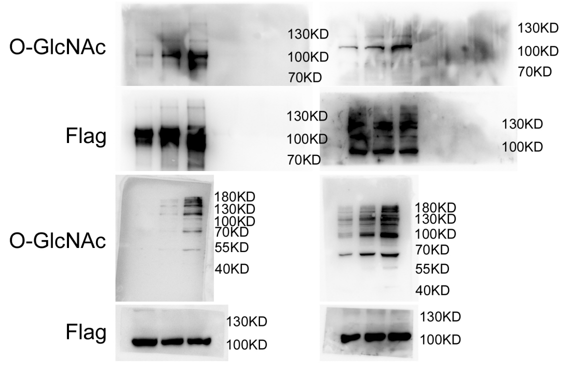

Fig S2I

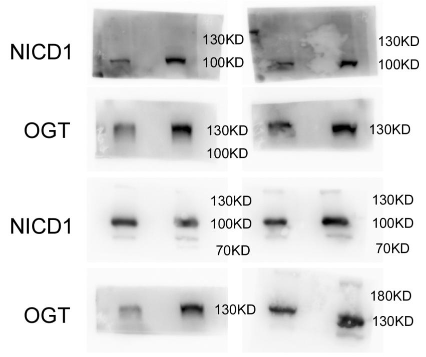

Fig S2J

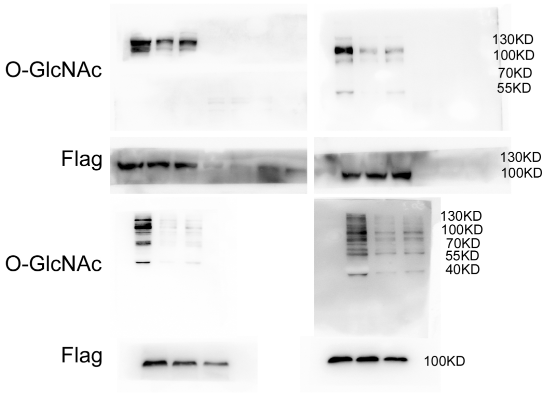

Fig S4A

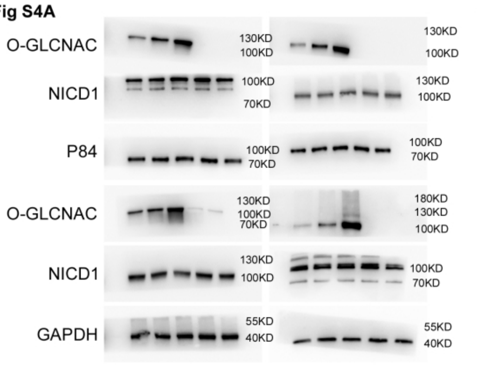

Fig S4B

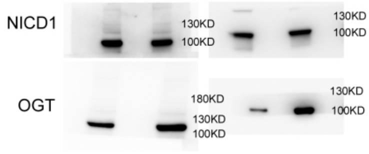

Fig S4C

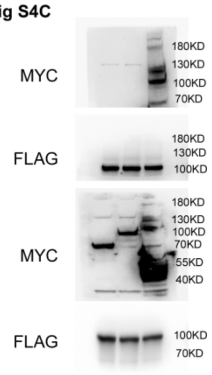

Fig S4E

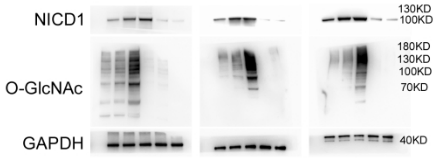

Fig S4F

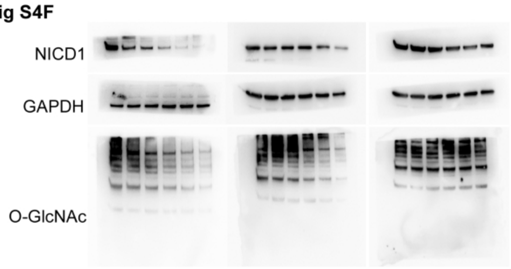

Fig S4G

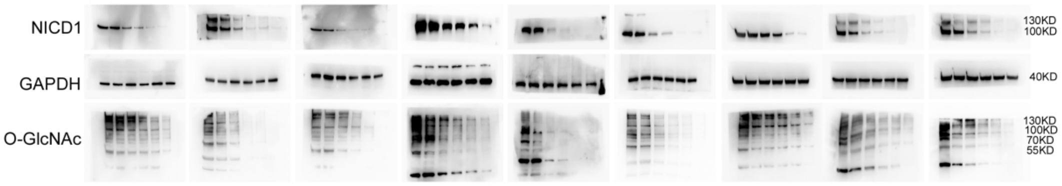

Fig S4H

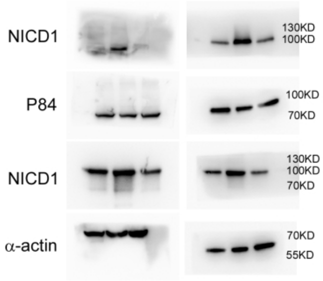

Fig S5A

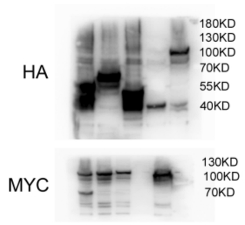

Fig S5C

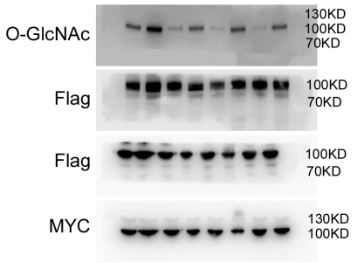

Fig S5D

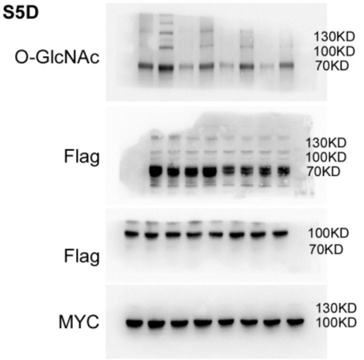

Fig S5G

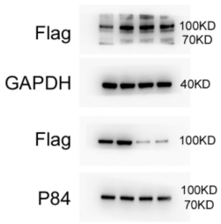

Fig S5I

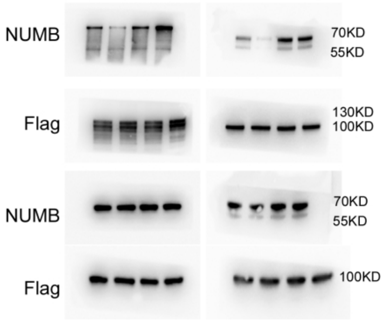

Fig S5J

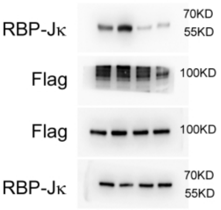

Fig S6G

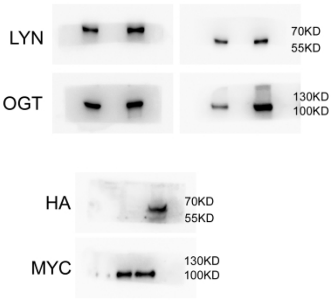

Fig S6H

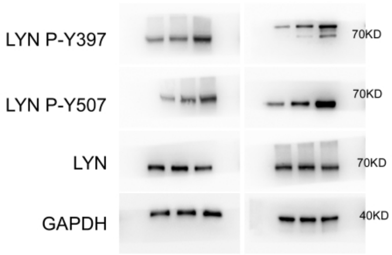

Fig S6K

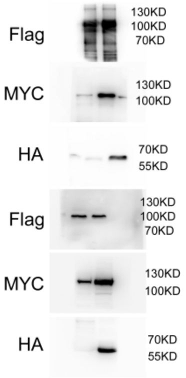

Fig S6J

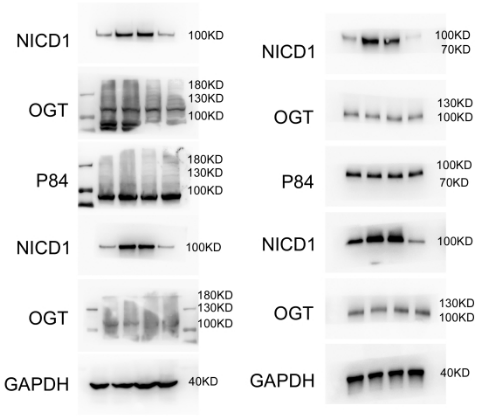

Fig S6L

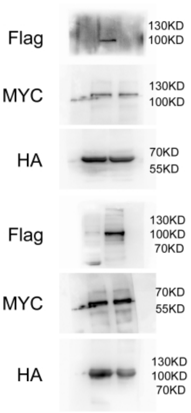

Fig S6M

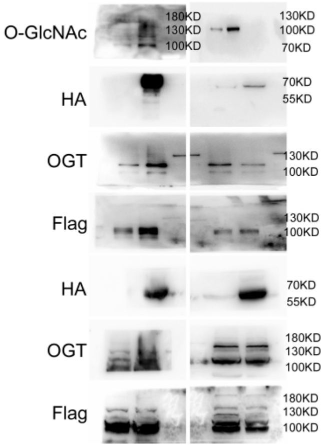

Fig S7B

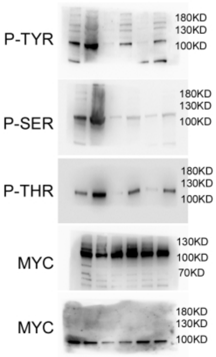

Fig S7C

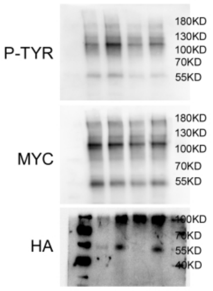

Fig S7D

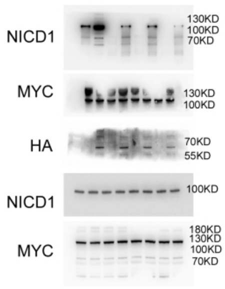

Fig S7J

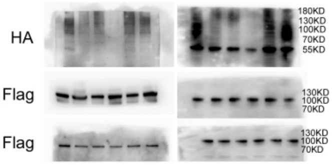

Fig S7I

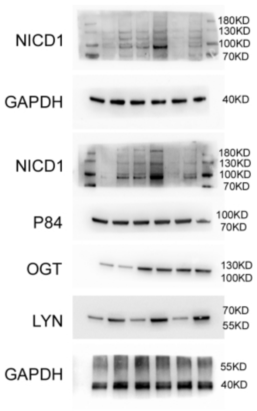

Fig S7K

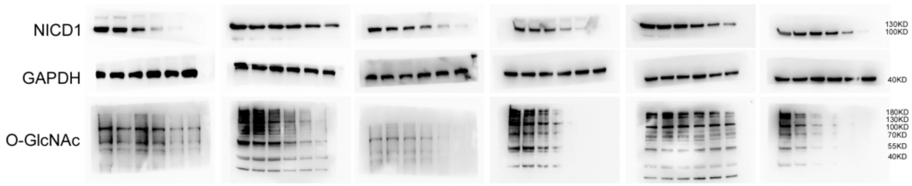

Fig S7L

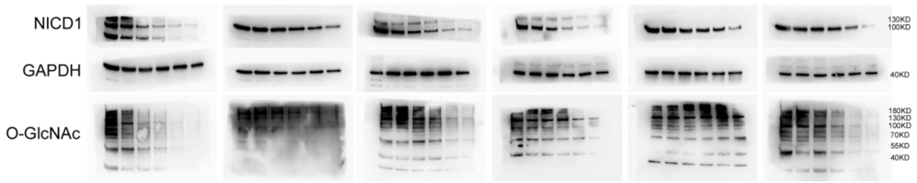

Fig S9F

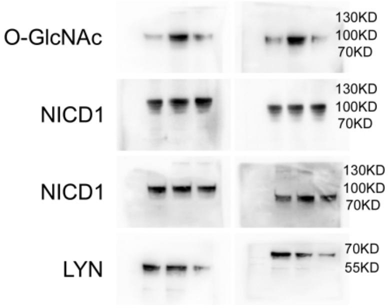

Fig S9I

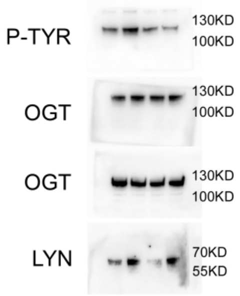

Fig S10B

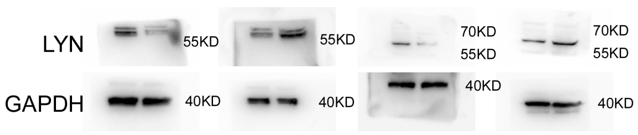

Supplement: Unedited blot and gel images [file jci-136-194378-s314.pdf]
